# Resveratrol and Curcumin in Stroke Therapy: From Experimental Evidence to Clinical Perspectives

**DOI:** 10.3390/nu18162713

**Published:** 2026-08-19

**Authors:** Mikołaj Grabarczyk, Aleksandra Szychowska, Weronika Szczepańska, Ewa Smolińska, Andrzej Glabinski, Piotr Szpakowski

**Affiliations:** 1 Department of Clinical Pharmacology, Medical University of Lodz, 90-153 Lodz, Poland; mikolaj.grabarczyk@stud.umed.lodz.pl; 2 The Nicolaus Copernicus Provincial Multispecialty Centre for Oncology and Traumatology in Lodz, 93-513 Lodz, Poland; alexbielenin@gmail.com; 3 J. Gromkowski Voivodship Specialist Hospital in Wroclaw, 51-149 Wroclaw, Poland; weronika.justynska@stud.umed.lodz.pl; 4 Central Teaching Hospital, Medical University of Lodz, 92-213 Lodz, Poland; ewa.smolinska@stud.umed.lodz.pl; 5 Department of Neurology and Stroke, Medical University of Lodz, Zeromskiego 113 Street, 90-549 Lodz, Poland; andrzej.glabinski@umed.lodz.pl

**Keywords:** polyphenols, resveratrol, curcumin, stroke, neurology

## Abstract

Stroke remains one of the leading causes of death and long-term neurological disability worldwide, while currently available therapeutic strategies are limited by narrow treatment windows and incomplete neuroprotection. In this context, plant-derived polyphenols have attracted increasing attention as potential adjunctive agents because of their multimodal biological activity. This review focuses on resveratrol and curcumin, two of the most extensively investigated polyphenols, and evaluates their potential role in the prevention and treatment of ischaemic and haemorrhagic stroke. Evidence from in vitro studies, animal models, and early clinical trials indicates that both compounds may attenuate key mechanisms involved in stroke-related brain injury, including oxidative stress, neuroinflammation, mitochondrial dysfunction, apoptosis, autophagy dysregulation, blood–brain barrier disruption, and microglial activation. Emerging evidence further suggests that interactions with the gut microbiota and modulation of the gut–brain axis may contribute to their biological effects by influencing intestinal barrier integrity, microbial metabolite production, systemic inflammation, and vascular risk. Preclinical studies show that resveratrol and curcumin can reduce infarct volume, limit cerebral oedema, preserve neuronal viability, promote angiogenesis and neurogenesis, and improve neurological and cognitive outcomes. Their beneficial effects have been reported both when administered before stroke onset and after cerebral injury, suggesting potential relevance for both prevention and post-stroke therapy. However, interpretation of these findings requires consideration of the translational limitations of experimental stroke models, which do not fully reproduce the heterogeneity, comorbidities, age profile, and variable reperfusion patterns characteristic of human stroke. Although commonly used models such as middle cerebral artery occlusion provide important mechanistic and therapeutic insights, preclinical efficacy should therefore not be regarded as a direct predictor of clinical benefit. Resveratrol and curcumin may also complement established and emerging treatment strategies, including thrombolysis, endovascular interventions, antihypertensive therapy, and stem cell-based approaches. Nevertheless, their clinical translation remains limited by poor solubility, low bioavailability, rapid metabolism, and insufficient clinical evidence. Novel formulations, including nanoparticles, exosome-based delivery systems, and structurally modified analogues, may help overcome these barriers by improving brain targeting and therapeutic efficacy. Overall, resveratrol and curcumin represent promising but still investigational candidates for adjunctive stroke therapy, requiring further well-designed clinical trials to define their optimal dosing, timing, safety, and clinical value.

## 1. Introduction

Phytochemicals are a diverse class of compounds, many of which have potential medical applications. Polyphenols are one of their most prominent representatives and comprise one of the largest groups of phytochemicals identified to date. More than 8000 distinct polyphenolic compounds have been described so far [1]. Structurally, they are characterised by the presence of aromatic rings bearing hydroxyl groups and are commonly divided into flavonoids, phenolic acids, lignans, and stilbenes [2,3]. In plants, polyphenols participate in protective and regulatory processes, whereas in humans their dietary intake has been associated with multiple health-promoting effects, largely attributed to their antioxidant and anti-inflammatory properties. However, evidence for direct therapeutic efficacy varies substantially between compounds, diseases, and clinical settings [3,4,5,6,7]. The medical relevance of polyphenols was initially discussed mainly in relation to cardiovascular health. Their intake has been associated with reduced blood pressure, improved lipid and glucose metabolism, and inhibition of platelet aggregation, all of which are relevant to vascular disease and thrombus formation [5,8]. More recently, increasing attention has been given to their potential neurological applications, including neuroinflammatory, neurodegenerative, epileptic, and tumour-related conditions [9,10,11,12,13,14,15,16,17]. These observations suggest that selected polyphenols may influence both vascular and neuronal mechanisms involved in cerebral injury. Resveratrol and curcumin are among the most widely studied plant-derived compounds with potential neuroprotective activity. Resveratrol is classified as a stilbene and can be extracted from peanuts, various berries, jackfruit, and eucalyptus. It is present at the highest concentrations in glycosidic forms, particularly in grape skins. Its chemical structure consists of two phenolic rings linked by a styrene double bond, forming 3,5,4′-trihydroxystilbene, with the trans-isomer being the most stable form of resveratrol. Resveratrol has a wide range of synthetic and natural analogues, adducts, derivatives, and conjugates. In human plasma, it typically occurs in glucuronide, sulphate, or free form. Owing to its distinct chemical structure, curcumin cannot be classified within any of the four major polyphenol subclasses. Therefore, a separate family, the curcuminoids, has been distinguished to classify this compound. The principal natural source of curcumin is turmeric, obtained from the rhizomes of *Curcuma longa*. Among the structural forms of curcumin found in plants are at least two tautomeric forms: keto and enol. The methoxy, hydroxyl, α,β-unsaturated carbonyl, and diketone groups that form the curcumin carbon skeleton are most likely responsible for its biological activity. In human plasma, curcumin occurs mainly as glucuronide and sulphate conjugates [18,19,20,21]. Beneficial properties of polyphenols are especially relevant in the context of stroke, a disorder in which vascular pathology, oxidative stress, inflammation, mitochondrial dysfunction, blood–brain barrier (BBB) disruption, and neuronal death interact to determine the extent of brain injury and functional recovery. Because resveratrol and curcumin may modulate several of these mechanisms simultaneously, they have attracted interest as potential adjunctive agents in stroke prevention and therapy [22,23,24,25,26]. This review summarizes current evidence from in vitro studies, animal models, and clinical trials regarding the role of resveratrol and curcumin in ischaemic and haemorrhagic stroke. The aim is to provide a parallel assessment of these two compounds as separate therapeutic candidates, rather than to propose a combined resveratrol-curcumin treatment strategy. Particular attention is given to their mechanisms of action, therapeutic timing, and potential combination with established or emerging treatments. The review also discusses limitations related to bioavailability, adverse effects, and cytotoxicity, as well as novel delivery systems designed to improve clinical translation.

## 2. Materials and Methods

### 2.1. Literature Search Strategy

Although this article is intended as a narrative review, a structured literature search was conducted to ensure a comprehensive overview of the current literature. The literature search was performed independently by two researchers, exclusively using the PubMed database. The primary objective was to identify relevant studies investigating the pharmacological properties and therapeutic potential of curcumin and resveratrol, with a particular focus on their neuroprotective effects during and after stroke. Additionally, a supplementary targeted search was conducted to evaluate the predictability and translational validity of animal stroke models.

### 2.2. Search Terms and Strings

Both researchers independently utilized the same specific keywords and Medical Subject Headings terms, combined using Boolean operators (AND, OR). The search strings included various combinations of the following terms: (“resveratrol” OR “curcumin” OR “polyphenols”) AND (“stroke” OR “ischemic stroke” OR “cerebral ischemia” OR “MCAO” OR “middle cerebral artery occlusion”) AND (“neuroprotection” OR “oxidative stress” OR “inflammation” OR “apoptosis”). For the supplementary search regarding experimental models, terms such as (“animal models” OR “experimental stroke” OR “translational research” OR “predictability”) were utilized.

### 2.3. Study Selection and Inclusion Criteria

Following their independent literature searches, the researchers collated their identified records into a single, consolidated database, and all duplicates were removed. The remaining list of articles was then screened based on titles and abstracts, followed by a full-text assessment to determine final eligibility. The inclusion criteria for the selected literature were:

- In vitro and in vivo preclinical studies (e.g., MCAO animal models) exploring the mechanisms of action of resveratrol and curcumin;
- Clinical trials and randomized controlled trials evaluating the efficacy and bioavailability of these polyphenols;
- Previously published review articles and meta-analyses providing background context;
- Methodological reviews and critical appraisals addressing the translational validity and limitations of experimental stroke models;
- Articles published in English up to the year 2026.

**Figure 1:**
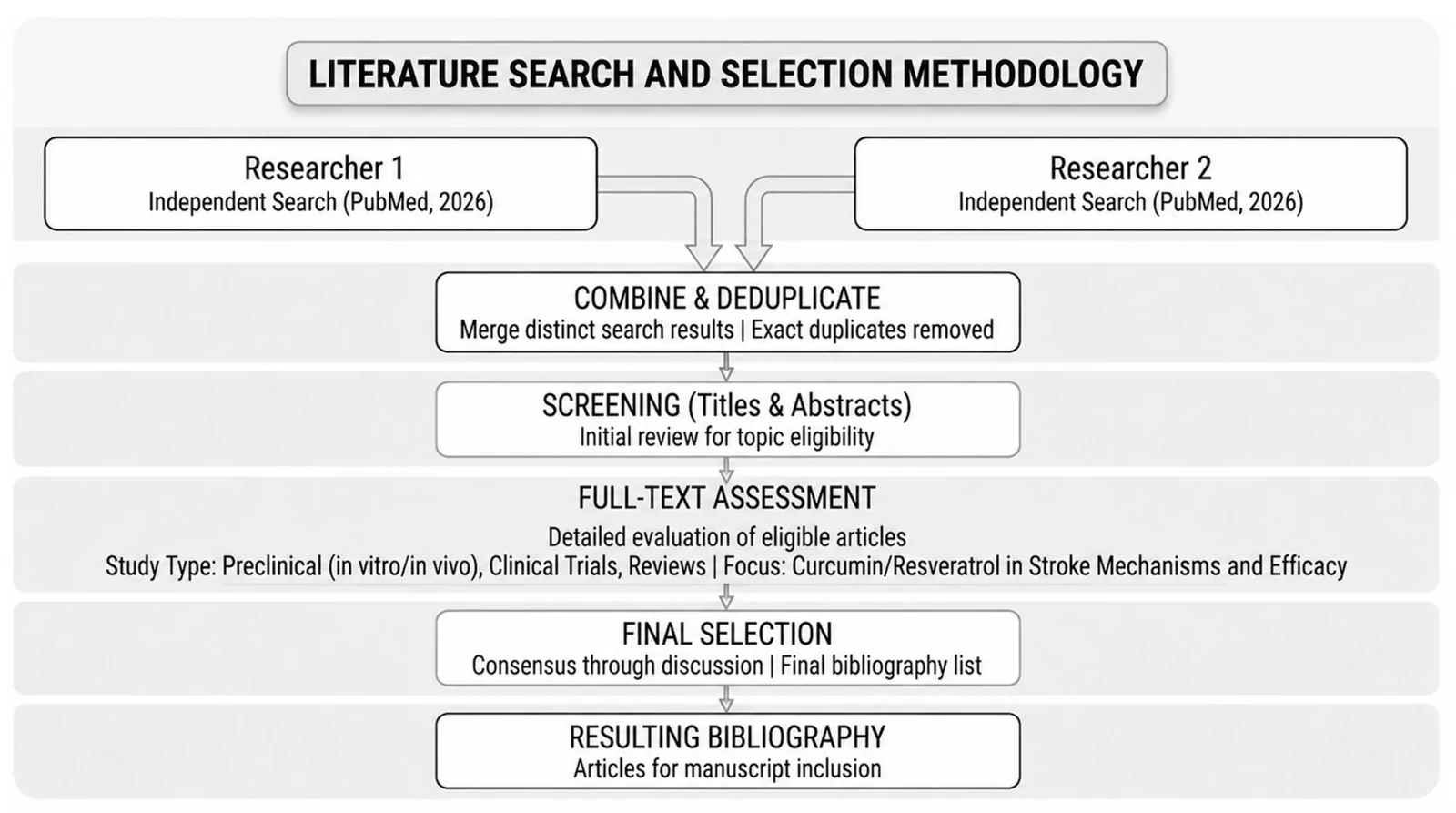
Flowchart of the selection of the papers for the review.

Studies lacking relevance to the neuroprotective, anti-inflammatory, or antioxidant mechanisms of resveratrol and curcumin, as well as those not published in English, were excluded from this review. Any discrepancies regarding the final inclusion of a specific article were resolved through mutual discussion to reach a consensus. Figure 1 shows the flowchart of the selection of the papers.

## 3. Stroke Types and Potential Place for Polyphenols in Their Treatment

Stroke is an acute neurological condition of the central nervous system (CNS), resulting from interruption of cerebral blood flow or rupture of an intracranial blood vessel. It leads to progressive brain damage as a result of hypoxia and reduced nutrient supply. The extent of injury depends on the size and location of the damaged or occluded vessel. The longer neurons and glial cells remain in an ischaemic state, the more extensive the resulting necrosis becomes. Although the consequences of stroke involve marked deterioration of neurological function, its underlying causes are predominantly cardiovascular. With approximately 12 million new cases and more than 6 million deaths each year, stroke remains the second leading cause of death attributable to a single disease [27,28]. Moreover, the burden of this condition has increased substantially over the past two decades, with the absolute number of stroke events rising by 70% and mortality by approximately 40%. The basic classification system distinguishes two main categories of stroke: ischaemic and haemorrhagic. Ischaemic stroke is the predominant type, accounting for approximately four out of five cases. The major risk factors for this condition include arterial hypertension, dyslipidaemia, diabetes, elevated body mass index (BMI), atrial fibrillation, and other prothrombotic states [27,28,29]. Standard treatment strategies include recombinant tissue plasminogen activators, such as alteplase and tenecteplase. These drugs act by dissolving the clot obstructing the artery, thereby restoring blood flow. Available therapeutic options also include emergency endovascular procedures aimed at mechanical recanalisation of the occluded vessels. Once the therapeutic time window has passed, the only available pharmacological intervention is acetylsalicylic acid, which is more effective in secondary prevention than in improving the patient’s immediate neurological condition [27,28,29,30]. Haemorrhagic stroke is more common in individuals with arterial hypertension and abnormalities of the vascular wall, such as aneurysms, lipohyalinosis, and cerebral amyloid angiopathy. Treatment is limited to controlling the bleeding and stabilising blood pressure. In cases of severe bleeding, neurosurgical intervention may be necessary to relieve pressure on brain tissue. Outcomes after stroke depend closely on the location and volume of the necrotic area. Residual symptoms reflect the functions of the lost neuronal populations. Neurological rehabilitation can partially restore motor and cognitive abilities, although its effectiveness depends largely on how early it is initiated [27,28,29,30]. To date, several neuroprotective substances that improve functional outcomes in patients after stroke have been identified and clinically evaluated. However, their use is still not recommended in official guidelines issued by scientific societies. Nevertheless, the search for new and more effective compounds continues. Polyphenols may be promising candidates for this role because of their low toxicity and multifaceted anti-inflammatory and antioxidant properties. The antioxidant activity of polyphenols involves numerous molecular targets at multiple levels of nervous cell metabolism. Restoration of mitochondrial function, upregulation of antioxidant enzyme levels, reduction in endoplasmic reticulum stress, and inhibition of lipid peroxidation are only a few examples of their potential to reduce stroke severity and promote neuronal recovery [31]. Polyphenols also stimulate the general xenobiotic response in target cells by activating multiple genes associated with defensive mechanisms. They exert neuroprotective effects by regulating factors that promote neuronal injury and by activating protective pathways that prevent neuronal death. Moreover, polyphenols influence epigenetic modulation, including DNA methylation and histone modification. These effects are mediated through interactions with histone deacetylases (HDACs) and DNA methyltransferases. Polyphenol-mediated neuroprotection is not limited to direct effects on neurons but also involves modulation of several immune cell populations within the CNS, including mast cells and microglia. Furthermore, their anticoagulant and antiplatelet activities may contribute to the prevention of thrombus formation. Taken together, these mechanisms provide a biological rationale for investigating polyphenols as potential adjunctive neuroprotective agents in stroke that may help broaden the therapeutic window in patients with stroke, accelerate recovery and improve post-stroke quality of life [32,33,34,35,36].

## 4. Translational Validity of Animal Models for Stroke Drug Discovery and Interventions

### 4.1. Principal Animal Models of Cerebral Ischemia

Animal models are widely used to investigate the pathophysiology and treatment of cerebral ischemia and currently constitute the main experimental source of evidence for evaluating the effects of polyphenols in stroke therapy, although none fully reproduces the heterogeneity of human stroke. Middle cerebral artery occlusion (MCAO) primarily models focal ischemic stroke, whereas bilateral common carotid artery occlusion (BCCAO) and four-vessel occlusion ischemia (4VOI) are mainly used to reproduce global cerebral ischemia or chronic cerebral hypoperfusion [37,38]. MCAO is considered the most clinically relevant rodent model of focal ischemic stroke because the middle cerebral artery is frequently involved in human anterior-circulation stroke. In the commonly used intraluminal filament technique, a filament is introduced through the carotid artery and advanced to block the origin of the MCA. Permanent MCAO models persistent vascular occlusion, whereas transient MCAO permits reperfusion after filament withdrawal and is therefore used to investigate ischemia–reperfusion injury [37,38,39]. Proximal occlusion usually damages both the cortex and striatum, while distal MCAO produces a predominantly cortical infarct. MCAO reproduces several characteristic features of human stroke, including focal cerebral blood flow reduction, formation of an ischemic core and penumbra, neurological deficits, edema, blood–brain barrier disruption, and neuroinflammation. However, filament MCAO causes an abrupt mechanical occlusion without thrombus formation, while reperfusion is rapid and experimentally controlled. It therefore differs from the variable and often incomplete recanalization observed in patients. Thromboembolic MCAO is more suitable for testing thrombolytic treatment but produces greater variability in clot localization, infarct size, and recanalization [37,38]. BCCAO involves temporary or permanent occlusion of both common carotid arteries. Its effects depend strongly on species and collateral circulation. In gerbils, poor posterior collateral supply results in severe forebrain ischemia and delayed hippocampal neuronal death. In rats and mice, vertebrobasilar circulation may maintain partial cerebral blood flow, increasing experimental variability [40]. Transient BCCAO is therefore more representative of global ischemia caused by cardiac arrest, severe hypotension, or systemic circulatory failure than of focal thromboembolic stroke. Permanent BCCAO produces chronic cerebral hypoperfusion, white-matter injury, and cognitive impairment, making it more relevant to vascular cognitive impairment and chronic carotid insufficiency [40]. The classic 4VOI model combines permanent occlusion of both vertebral arteries with subsequent transient occlusion of both common carotid arteries. It causes severe forebrain ischemia followed by reperfusion and characteristic delayed loss of hippocampal CA1 neurons [41,42,43]. The model is particularly useful for studying selective neuronal vulnerability, cognitive dysfunction, and neuroprotective mechanisms. However, like transient BCCAO, it corresponds more closely to global ischemia after cardiac arrest than to typical focal stroke. It does not reproduce focal arterial occlusion, a spatially defined ischemic penumbra, or lateralized neurological deficits. Overall, MCAO provides the closest approximation of common focal ischemic stroke, especially anterior-circulation large-vessel occlusion. BCCAO and 4VOI are more appropriate for studying chronic hypoperfusion and global cerebral ischemia, respectively. Nevertheless, translation remains limited because experimental studies commonly use young, healthy animals, whereas stroke predominantly occurs in older patients with hypertension, diabetes, atherosclerosis, and other comorbidities. Model selection should therefore reflect the specific clinical mechanism being investigated, and results should ideally be confirmed using aged or comorbid animals and more than one ischemia model [44,45].

### 4.2. Translational Relevance of Animal Models of Ischemic Stroke

The predictive value of animal models is supported by several successful examples of translation into clinical practice. Recombinant tissue plasminogen activator (rtPA), currently the standard pharmacological treatment for acute ischemic stroke, demonstrated efficacy in experimental thromboembolic models prior to its clinical application, illustrating that appropriately selected animal models can predict therapeutic responses observed in humans [46,47]. Importantly, preclinical studies not only predicted the beneficial effects of rtPA on neurological outcomes but also identified an increased risk of hemorrhagic complications and a progressive decline in efficacy with treatment delay, findings that were subsequently confirmed in clinical practice and contributed to the concept that “time is brain” in acute stroke management [48]. Additional examples of successful translation include statin therapy and endovascular thrombectomy. A meta-analysis of 41 experimental stroke studies demonstrated that statin treatment significantly reduced infarct volume and improved neurological outcomes in animal models [49]. These findings are consistent with the results of the SPARCL trial, which demonstrated that intensive atorvastatin therapy reduced the risk of recurrent stroke in patients with a recent stroke or transient ischemic attack [50]. Similarly, experimental stroke models have contributed to the understanding of reperfusion mechanisms and played an important role in the development and optimization of thrombectomy devices. Preclinical studies enabled the assessment of clot–device interactions, recanalization efficacy, and procedural safety before clinical implementation, while subsequent clinical trials established mechanical thrombectomy as a standard treatment for acute ischemic stroke caused by large-vessel occlusion [51,52,53]. Despite these successes, the translation of experimental findings into effective clinical therapies remains challenging. Numerous neuroprotective strategies have demonstrated beneficial effects in experimental stroke models, yet only a limited number have translated into effective clinical interventions. This discrepancy has been attributed to differences between experimental and clinical settings, including the use of young, healthy animals, the absence of common human comorbidities, and differences in treatment timing and outcome assessment [46,47]. Therefore, findings obtained in experimental stroke models should not be viewed as direct predictors of clinical efficacy, but rather as an important step in the translational pathway from basic research to human studies. Nevertheless, animal stroke models remain at the forefront of efforts to understand stroke pathophysiology and provide valuable preclinical data guiding future clinical trials. Recent evidence suggests that the translational value of preclinical findings depends not only on the investigated therapy but also on the choice of experimental model, with larger animal models potentially offering greater clinical relevance than traditional rodent models [54]. In this context, the beneficial effects of resveratrol and curcumin observed in experimental stroke models should be considered biologically relevant and mechanistically informative, while their clinical utility requires confirmation in well-designed human studies.

## 5. Resveratrol in Stroke-Related Neuroprotection: Experimental and Clinical Evidence

### 5.1. Evidence from In Vitro Studies

**Figure 2:**
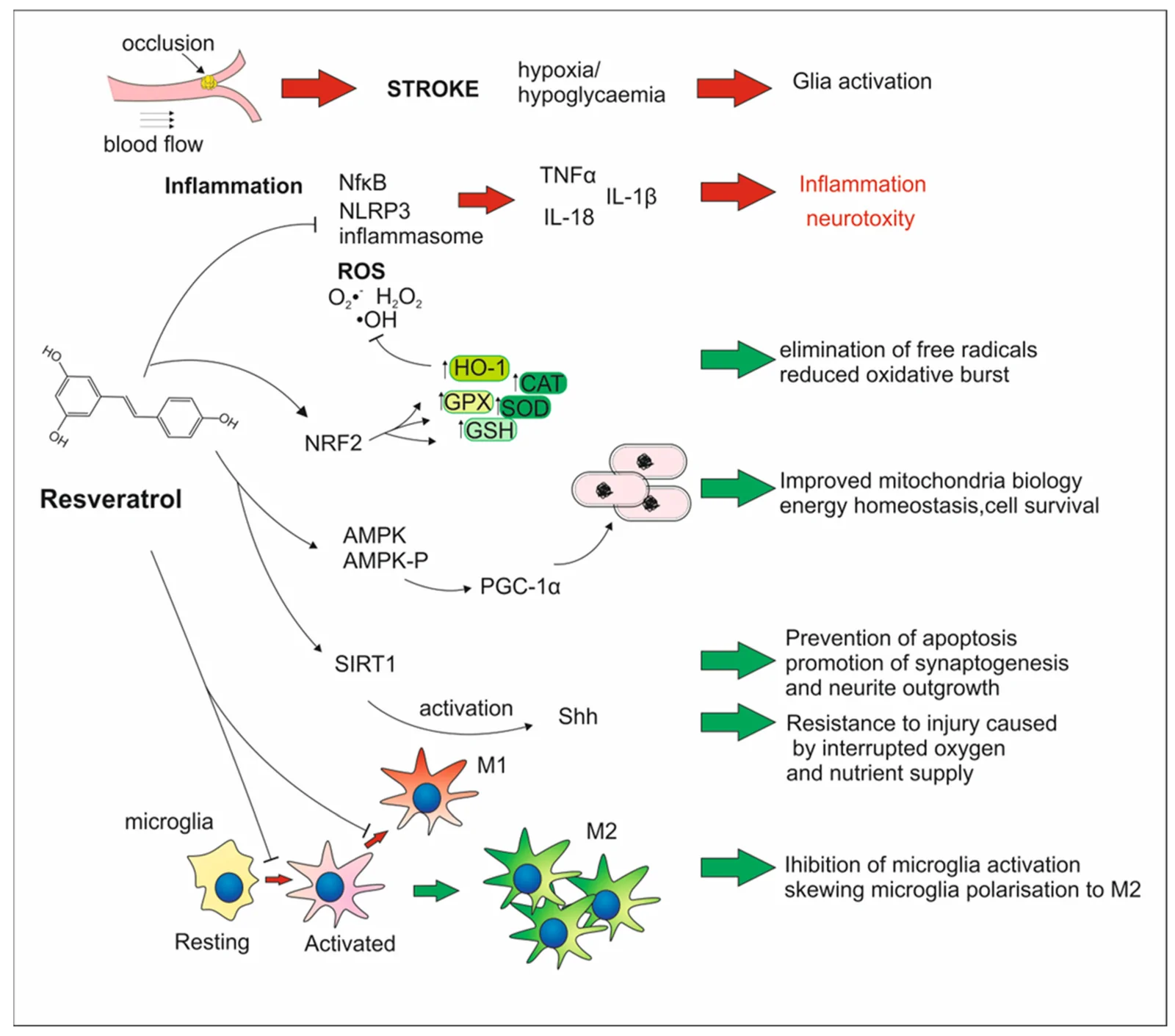
Summarised molecular mechanisms underlying the neuroprotective effects of resveratrol in experimental stroke. Cerebral ischemia induces oxidative stress, activation of NF-κB/NLRP3 inflammasome signalling, mitochondrial dysfunction, and microglial activation, leading to enhanced neuroinflammation, apoptosis, and neuronal injury. Resveratrol counteracts these pathological processes by activating NRF2-dependent antioxidant responses, increasing the expression of antioxidant enzymes (HO-1, SOD, CAT, GPX and GSH), improving mitochondrial function through AMPK/PGC-1α signalling, stimulating SIRT1 and Shh pathways, and promoting microglial polarization toward the anti-inflammatory M2 phenotype. Together, these mechanisms contribute to reduced oxidative stress, attenuation of neuroinflammation, enhanced neuronal survival, and improved functional recovery after stroke.

The in vitro oxygen-glucose deprivation (OGD) model provides a valuable platform for improving our understanding of neuronal injury induced by hypoxia and hypoglycaemia, as well as for evaluating new therapeutic strategies for cerebral ischaemic damage. Cellular injury caused by hypoxic conditions involves activation of the nuclear factor kappa B (NF-κB) pathway and the NLR family pyrin domain containing 3 (NLRP3) inflammasome, a critical mediator of ischaemia-induced inflammation, which together initiate a detrimental cycle of oxidative stress ultimately leading to neurotoxicity [55,56]. A study using a three-dimensional gelatin scaffold culture of neuronal SH-SY5Y cells demonstrated that resveratrol inhibits activation of both NF-κB and the NLRP3 inflammasome, thereby reducing the production of inflammatory cytokines, including tumour necrosis factor alpha (TNF-α), interleukin-1 beta (IL-1β), and interleukin-18 (IL-18). In addition, this polyphenol appears to reduce reactive oxygen species (ROS) levels and, consequently, oxidative stress, potentially via activation of nuclear factor erythroid 2-related factor 2 (NRF2) and upregulation of its downstream antioxidant target genes, such as superoxide dismutase (SOD), glutathione peroxidase (GPx), glutathione (GSH), catalase (CAT), and heme oxygenase 1 (HO-1). NRF2 plays a pivotal role in cellular defence mechanisms and in the activation of survival pathways in response to oxidative stress. Its expression is typically reduced in stroke-affected tissues [55]. 5′ adenosine monophosphate-activated protein kinase (AMPK) acts as a neuroprotective regulator that maintains energy homeostasis, metabolism, and cell viability. In a study conducted by Chien-Hung Lin et al., resveratrol was shown to increase the expression of AMPK and phosphorylated AMPK in human SH-SY5Y neuronal cells subjected to OGD. This in vitro model is also associated with mitochondrial dysfunction, characterised by a decrease in D-loop levels, mitochondrial mass, maximal respiratory capacity, cytochrome c oxidase (COX) activity, and mitochondrial membrane potential. All of these alterations can be counteracted, at least to some extent, by the addition of resveratrol to the culture medium. Moreover, such intervention is associated with upregulation of peroxisome proliferator-activated receptor gamma coactivator 1 α, as well as increased expression of nuclear respiratory factor 1 and mitochondrial transcription factor A [56]. Other protective mechanisms initiated by resveratrol include activation of the sonic hedgehog (Shh) signalling pathway, which may prevent apoptosis and enhance synaptogenesis and neurite outgrowth in hypoxic neurons [57]. Resistance to injury induced by interruption of oxygen and nutrient supply can be increased by exposing tissues to short, repeated periods of ischaemia in a process known as ischaemic preconditioning (IPC). One of the key molecular factors responsible for the effectiveness of this process is sirtuin 1 (SIRT1). Research performed by Raval et al. on organotypic hippocampal slice cultures demonstrated that pretreatment with resveratrol induces a neuroprotective effect similar to that of IPC, likely because of its ability to stimulate SIRT1 [58]. Several studies have indicated that the pharmacological properties of resveratrol depend on a number of factors, including its concentration, the method and timing of administration, the physiological or pathological state of the tissue, and the specific cell or organism type involved. Under some conditions, its effects on biological systems are not necessarily beneficial. At concentrations exceeding 50 μM in culture medium, resveratrol was observed not only to trigger SIRT1-independent activation of AMPK, but also to induce toxic effects, such as a significant decline in mitochondrial membrane potential and cellular adenosine triphosphate (ATP) levels in C2C12 cells [59]. Furthermore, Pineda-Ramírez et al. reported that primary neuronal cultures derived from rat brains exhibited exacerbated histological injury associated with induced excitotoxicity when exposed to resveratrol at a concentration of 400 μM [60]. However, considering the pharmacokinetic properties of polyphenols, such concentrations would be extremely difficult to achieve in CNS tissue fluids. Moreover, these compounds may exert beneficial effects at concentrations far lower than the reported harmful ones, which may help overcome this potential obstacle in translation to clinical use. The pleiotropic neuroprotective mechanisms of resveratrol, including its antioxidant, anti-inflammatory, anti-apoptotic and microglia-modulating effects, are summarized in Figure 2.

### 5.2. Evidence from Animal Models

#### 5.2.1. Administration of Resveratrol After the Onset of Ischemia

A substantial body of evidence from numerous animal studies has consistently demonstrated the neuroprotective potential of resveratrol in a variety of stroke models. Zhang et al. observed that administration of resveratrol after the onset of cerebral ischaemia improves neuronal function, reduces infarct volume, and preserves primary neuronal structure in rats. These effects are partly mediated by suppression of the cluster of differentiation 147/matrix metalloproteinase 9 (MMP-9) pathway, which attenuates activation of the pro-inflammatory response and protects against brain injury [61]. Moreover, resveratrol reduces neuroinflammation not only by inhibiting microglia, the principal effector cells of the immune system within the CNS, but also by shifting their polarisation from the pro-inflammatory M1 phenotype to the anti-inflammatory M2 phenotype. This effect is associated with downregulation of microRNA-155 and of M1-associated markers, including CD32 and IL-1β. At the same time, resveratrol promotes the expression of M2-associated transcripts, such as CD206 and arginase [62]. Dong et al. reported that post-stroke administration of resveratrol reduces infarct volume and promotes neurological recovery, largely through stimulation of angiogenesis, as evidenced by increased expression of matrix metalloproteinase 2 (MMP-2) and vascular endothelial growth factor (VEGF), one of the key mediators of blood vessel growth. These beneficial effects are more pronounced when the phytochemical is administered during the early stages of the ischaemia–reperfusion phase, with significant improvements observed seven days after middle cerebral artery occlusion/reperfusion (MCAO/R) [63]. In animal models, resveratrol has also been associated with attenuation of cerebral oedema, a common complication of brain ischaemia. This effect, together with reduction in infarct volume and BBB disruption, appears to be mediated through suppression of transcription factors of the Sp family. Another important mechanism of action is the reduction in sulfonylurea receptor 1 (SUR1) and aquaporin 4 expression, both of which are associated with oedema formation [64]. Resveratrol has also been reported to reduce markers of brain injury and inflammation in rodent models of mild ischaemic injury and recurrent stroke. Moderate doses of this phytochemical administered to rats within 3 days after the onset of cerebral ischaemia lead to reduced BBB leakage and inflammation, as well as improved endothelial protection. These responses are at least partly mediated by SIRT1-dependent mechanisms. However, no significant effects have been observed on regional cerebral blood flow, blood pressure, or core temperature [65]. In models of the second main type of stroke, namely intracerebral haemorrhage (ICH), resveratrol has likewise been shown to exert neuroprotective effects by modulating the inflammatory response. Sun et al. reported that this polyphenol improves neurological function, reduces cell death, and facilitates haematoma clearance, partly by inhibiting activation of CD16+ macrophages/microglia and tumour necrosis factor alpha release. Findings obtained in mice genetically modified to lack expression of sirtuin 3 (SIRT3) suggest that this anti-inflammatory effect may be linked to modulation of the SIRT3 signalling axis [66].

#### 5.2.2. Administration of Resveratrol Prior to the Onset of Ischemia

One of the earliest reports investigating the potential of resveratrol as a protective agent against acute ischaemic stroke was published by Sinha et al. The authors observed that administration of trans-resveratrol for 21 days prior to middle cerebral artery occlusion (MCAO) significantly improved subsequent motor performance and reduced infarct volume in rats [67]. Supporting these findings, Ren et al. demonstrated that intraperitoneal administration of resveratrol for 7 days before MCAO attenuates cerebral ischaemic damage and alleviates the resulting neurological impairment. This study indicated that the neuroprotective effect is likely mediated through upregulation of NRF2 and HO-1, which help limit oxidative damage, as well as through reduced expression of caspase-3. This mechanism becomes evident as early as 2 h after ischaemia and reaches its peak within 24 h, highlighting the importance of the antioxidant response element pathway in the treatment of the acute phase of stroke [68]. Additional studies using rodent models of cerebral ischaemia have shown that resveratrol may act as an agonist of peroxisome proliferator-activated receptor alpha and gamma (PPAR-α and PPAR-γ). Moreover, this mechanism appears to be crucial for its neuroprotective effects, as mice lacking PPAR expression do not show significant neurological benefit from resveratrol pretreatment [69]. Preservation of cholinergic signalling in memory-related brain regions affected by impaired blood flow is another important mechanism underlying resveratrol-induced neuroprotection. Administration of this phytochemical contributes to maintenance of cholinergic cell viability in the basal forebrain, which may mitigate cognitive decline following cerebral ischaemia [70]. Another important mechanism of action is inhibition of apoptosis. Suppression of programmed cell death may increase neuronal survival within the ischaemic penumbra. This effect is at least partly attributable to resveratrol-mediated activation of the Janus kinase 2/signal transducer and activator of transcription 3 (STAT3) and phosphoinositide 3-kinase (PI3K)/Akt/mammalian target of rapamycin (mTOR) pathways [71]. Among the signalling pathways involved, researchers also highlight the Shh axis. Animals pretreated with resveratrol for 7 days before induction of cerebral artery occlusion show increased expression of sonic hedgehog, patched-1 (Ptc-1), smoothened (Smo), and glioma-associated oncogene 1 (Gli-1) mRNAs, accompanied by translocation of Gli-1 to the nucleus. These molecular changes are associated with reduced infarct volume, increased cell viability in necrotic areas, and improved functional recovery [72]. The attenuation of inflammatory responses in microglial cells also appears to depend on modulation of Shh-related mechanisms. This process involves relocation of Smo to the primary cilium and enhancement of Shh-dependent signal transduction [73]. The neuroprotective effects of preconditioning with this polyphenol are strongly dependent on the timing of pretreatment. Current research indicates that repetitive preconditioning with resveratrol (RPC) induces the most effective ischaemic tolerance when initiated no later than 2 days before injury [58,74]. Nevertheless, it is worth noting that a single administration before MCAO is sufficient to induce at least partial protection. Some authors suggest that SIRT1 may mediate this tolerance through upregulation of brain-derived neurotrophic factor (BDNF) and downregulation of uncoupling protein 2 (UCP2) [75]. Della-Morte et al. demonstrated that repetitive intraperitoneal injections of resveratrol mimic IPC, thereby protecting the cornu ammonis 1 region of the hippocampus during global cerebral ischaemia. This protection depends on activation of SIRT1, a NAD+-dependent deacetylase associated with lifespan extension induced by caloric restriction. Enhanced mitochondrial ATP synthesis and stimulation of microRNA-149-5p, a key regulator of caspase-3-mediated apoptotic neuronal cell death, also appear to be involved [74,76]. A different perspective on this phenomenon focuses on the mechanisms through which RPC facilitates prolonged ischaemic tolerance, emphasising improved metabolic and bioenergetic efficiency rather than metabolic depression alone. RNA sequencing performed on mouse cortices revealed that RPC initially suppresses mitochondrial respiration, thereby triggering adaptive cellular responses. These responses are characterised by epigenetically coupled mitochondrial and nuclear adaptations of acetyl-coenzyme A metabolism and lead to increased baseline ATP levels. In turn, this elevated energy state delays the onset of excitotoxic injury [77]. A recent study by Jackson et al. identified a novel neuroprotective mechanism of resveratrol pretreatment involving prevention of ischaemia-induced poly(ADP-ribose) polymerase 1 (PARP-1) overexpression and PARP-1-mediated cell death, known as parthanatos, in ischaemic brain tissue [78]. The anti-inflammatory properties of resveratrol also show promising, although limited, effects in stroke prevention and attenuation in animal models. IL-1β plays a key role in accelerating stroke onset in stroke-prone spontaneously hypertensive rats (SHRSP). Dietary restriction (DR) has been shown to reduce inflammation and significantly delay the onset of cerebral ischaemia in these animals [79,80]. Resveratrol mimics some of the transcriptional changes induced by DR, with beneficial effects reflected in improved insulin sensitivity, cardiovascular function, bone density, motor coordination, delayed cataract formation, and systemic anti-inflammatory activity. The latter is particularly evident in a marked reduction in IL-1β-induced monocyte chemoattractant protein 1 mRNA expression and in decreased nuclear levels of the inflammatory proteins p50 and p65. Unfortunately, unlike DR, resveratrol does not prolong the lifespan of either healthy or SHRSP animals during short observation periods [81,82]. Results from in vivo studies regarding resveratrol’s role in treatment of stroke are summarized in Table 1.

### 5.3. Resveratrol Formulations and Structural Modifications Designed to Improve Bioavailability and Target-Tissue Delivery

Despite the beneficial effects of resveratrol in mitigating ischaemia/reperfusion injury, its limited solubility and short half-life restrict its clinical applicability. Among the available methods for improving the pharmacokinetics and bioavailability of resveratrol, nanoparticle-based delivery systems have attracted particular attention. Biodegradable resveratrol nanoparticles (RES-NPs), obtained using poly(N-vinylpyrrolidone)-b-poly(ε-caprolactone), have been investigated as a strategy to enhance protection against oxidative stress in rat cortical cells. These RES-NPs exhibit controlled release, neuronal compatibility, and more efficient endocytic uptake by neurons. Compared with free resveratrol, RES-NPs provide superior neuroprotection, as indicated by reduced lactate dehydrogenase (LDH) release, reactive oxygen species activity, malondialdehyde (MDA) levels, and changes in the profile of apoptosis-related markers [84]. Nanoparticles coated with polysorbate 80, developed by Ashafaq et al., show improved neuronal permeability and significantly enhanced brain targeting. This type of formulation also markedly amplifies the antioxidant and anti-apoptotic properties of resveratrol, with reported potency ranging from 800- to 3000-fold greater than that of free resveratrol. Analyses performed in stroke models indicate that these effects are dose-dependent, with stronger neuroprotection achieved at higher doses [85]. Controlled, ROS-responsive release of resveratrol can be achieved by conjugating the compound with polylactic acid (PLA)-coated mesoporous silica nanoparticles containing low-density lipoprotein receptor (LDLR) ligand peptides. Drug release is triggered by ROS, as PLA degradation accelerates under oxidative conditions, whereas LDLR ligands enhance transcytosis across the BBB [86]. A system designed by Wang et al. employs pH-responsive polyethylene glycol-acetal-polycaprolactone micelles loaded with resveratrol. This formulation also improves BBB penetration and enables mitochondrial targeting as the intended intracellular destination of the compound [87]. Further innovations include the macrophage membrane-coated nanoparticles developed by Su et al., which integrate resveratrol, curcumin, and neuroprotective sialic acid-modified polysaccharides. These nanoparticles demonstrate superior photostability, BBB penetration, and neuroprotective efficacy compared with free resveratrol, significantly reducing cerebral infarct size and improving neurological outcomes in animal models of stroke [88]. An alternative approach to enhancing the bioavailability of resveratrol and modifying its pharmacological potential involves the use of potent and stable resveratrol analogues. Malibatol A (MA), a resveratrol oligomer, significantly improves neurological scores, reduces infarct volume, and decreases levels of oxidative stress markers, including ROS, 3-nitrotyrosine, and 4-hydroxynonenal, within 24 h after cerebral injury. Furthermore, MA preserves mitochondrial enzyme activity and stabilises mitochondrial membrane potential. It also exerts anti-apoptotic effects by increasing B-cell lymphoma 2 (Bcl-2) expression, reducing Bcl-2-associated X protein (Bax) levels, preventing cytochrome c release, and inhibiting activation of caspases 3 and 9 [89]. The resveratrol tetramer vaticanol C activates peroxisome proliferator-activated receptor alpha (PPAR-α) and peroxisome proliferator-activated receptor beta/delta (PPAR-β/δ) pathways, thereby modulating inflammation and metabolism in affected tissues and exerting protective effects. Another oligomer, ε-viniferin, shows antioxidant activity similar to that of resveratrol, but does not affect PPARs or SIRT1 [90]. Interestingly, vaticanol C acts as a dual activator of PPAR-α and PPAR-β/δ, whereas resveratrol itself activates all three PPAR subtypes, namely α, β/δ, and γ. These findings highlight how structural variation among phytochemical analogues can influence receptor interactions and therapeutic potential [90]. Modified forms of resveratrol may also prove useful in prevention. Hybrid compounds generated by combining tetramethylpyrazine with resveratrol have been shown to inhibit platelet aggregation induced by adenosine diphosphate (ADP) or arachidonic acid. Available analyses indicate that their anti-aggregatory and antioxidant effects are superior not only to those of the parent compounds, but also to those of reference drugs such as ticlopidine and aspirin [91]. Reports from studies assessing the bioavailability and effectiveness of resveratrol particles modified with chemical alterations or carrier compounds are summarized in Table 2.

### 5.4. The Enhancing Potential of Resveratrol on Other Therapy Forms

Resveratrol appears to exert a synergistic effect when combined with α-lipoic acid. The beneficial effects of this combination are considerable, as substantial neuroprotection can be achieved even with subtherapeutic doses of resveratrol. Experimental studies indicate that the optimal ratio of the two compounds is 1:1 resveratrol to α-lipoic acid. This proportion provides a level of neuroprotection comparable to that achieved with 100-fold higher doses of free, unmodified resveratrol. However, this treatment must be administered within a specific therapeutic window, as beneficial outcomes have been observed only in rodent models receiving the drug combination during cerebral artery occlusion [83]. The combination of resveratrol with the histone deacetylase inhibitor MS-275 (entinostat) has also demonstrated significant neuroprotective effects following transient MCAO and OGD. Previous research has shown that histone deacetylation and altered acetylation of NF-κB/RelA, particularly at lysine 310, are characteristic features of ischaemic brain injury [92,93]. Lanzillotta et al. demonstrated that resveratrol and entinostat act synergistically to confer neuroprotection in a mouse model of ischaemic stroke by restoring physiological acetylation patterns. Resveratrol activates the AMPK-SIRT1 pathway, whereas MS-275 inhibits class I HDAC activity. This combined approach normalises RelA acetylation, leading to a shift in RelA binding from the pro-apoptotic Bcl-2-interacting mediator of cell death gene to the anti-apoptotic B-cell lymphoma-extra large gene. In addition, the activity of the nitric oxide synthase 2 (NOS2) promoter is reduced, resulting in a broad anti-inflammatory effect reflected by downregulation of markers such as NOS2, interleukin-6 (IL-6), IL-1β, mannose receptor C-type 1, and Ym1, as well as by reduced leukocyte infiltration. Furthermore, this treatment promotes markers of alternative microglia/macrophage activation, including arginase 1 (Arg-1), Ym1, and Fc fragment of immunoglobulin G receptor IIb, while also restoring normal microglial morphology [92,94]. At the anatomical and functional levels, these effects are reflected in reduced infarct volume and significant alleviation of neurological deficits caused by cerebral ischaemia [92]. To enhance the translational potential of this strategy, Faggi et al. investigated the possibility of replacing MS-275 with valproate, which also inhibits class I HDACs. In neurons exposed to OGD, valproate exerts neuroprotective effects, and its pharmacologically effective dose is lower when it is combined with resveratrol. This combination restores histone acetylation and produces favourable outcomes in a transient MCAO model, indicating a promising therapeutic strategy for post-ischaemic brain injury [95]. Liu et al. demonstrated that in a MCAO/R rat model, daily pretreatment with rosuvastatin and resveratrol for 7 days before the onset of cerebral ischaemia results in significantly improved neurological outcomes, reduced infarct size, decreased caspase-3 and IL-1β levels, as well as increased Bcl-2/Bax and microtubule-associated protein 1 light chain 3 II/I (LC3II/LC3I) ratios. These findings indicate that the combined treatment is more effective than either agent alone [96]. Endovascular thrombectomy is the preferred treatment for strokes caused by large artery occlusion. However, it has been shown to induce oxidative stress, disrupt the BBB, trigger apoptosis, and impair neurogenesis as adverse consequences of rapid reperfusion. In experimental settings, intra-arterial delivery of resveratrol-loaded polymeric nanoparticles has been proposed as a strategy to attenuate reperfusion-related injury after intravascular intervention. In a transient MCAO rat model, this treatment significantly reduces oxidative stress, brain oedema, and apoptosis, while promoting neurogenesis through increased BDNF expression. However, its safety, feasibility, and efficacy in patients remain untested [97]. In rodents, resveratrol has been reported to augment recovery-related effects of neuronal progenitor cell transplantation by improving mitochondrial function and facilitating neuronal differentiation [98]. Resveratrol also has the potential to stimulate proliferation of neural stem cells following oxygen-glucose deprivation/reoxygenation (OGD/R) injury in vitro. In addition, it can induce differentiation of bone marrow-derived mesenchymal stem cells into neuron-like cells through activation of the Shh signalling pathway [99,100]. This stimulation of NSC proliferation via the Shh pathway increases cell survival in OGD/R injury models [99]. Similarly, Shanan et al. found that resveratrol combined with pyrroloquinoline quinone facilitates neurite outgrowth and increases cerebellar granule neuron viability [101].

### 5.5. Evidence from Clinical Trials

Clinical evidence regarding the efficacy of resveratrol in ischemic stroke remains limited, with only a few studies conducted to date. Recombinant tissue plasminogen activator remains the standard treatment for ischaemic stroke; however, its efficacy is limited by a narrow therapeutic window [102]. To address this limitation, Chen et al. investigated the potential of resveratrol to improve outcomes associated with delayed tissue plasminogen activator administration. In this study, 312 patients were divided into groups according to onset-to-treatment time: an early-onset group (0–120 min) and a delayed-onset group (120–240 min). Participants in both groups received tissue plasminogen activator (0.9 mg/kg) either with placebo or in combination with resveratrol (2.5 mg/kg; maximum dose 250 mg). In both the early- and delayed-onset groups, the addition of resveratrol resulted in significantly better outcomes compared with tissue plasminogen activator alone. This improvement was associated with reduced plasma levels of MMP-2 and MMP-9, as well as better scores on the National Institutes of Health Stroke Scale (NIHSS). These findings suggest that resveratrol may enhance the efficacy of tissue plasminogen activator therapy, particularly when treatment is delayed [102]. Although these findings are clinically relevant, they should be interpreted as preliminary and hypothesis-generating. They do not establish resveratrol as a validated adjunct to thrombolysis, and replication in independent, adequately powered randomized trials is required. Stroke is associated with numerous risk factors, including hypertension, dyslipidaemia, diabetes, obesity, and stress. Fodor et al. investigated the potential of resveratrol as a long-term strategy for secondary prevention in patients who had experienced a stroke within the previous year. In their study of 228 patients, resveratrol was administered in dose-dependent regimens (100 mg and 200 mg), resulting in significant reductions in blood pressure, BMI, and glucose levels, along with improvements in lipid parameters over a 12-month period. The 200 mg dose appeared to be more effective, particularly in reducing triglyceride levels. Notably, no new vascular events occurred in any of the treatment groups, suggesting that resveratrol may warrant further investigation as a complementary strategy in secondary prevention. However, the available data are insufficient to establish a reduction in recurrent vascular events or to support routine clinical use [103]. Moreover, not all clinical studies have demonstrated beneficial effects of this polyphenol. In a randomized, double-blind, placebo-controlled trial, patients received 510 mg of resveratrol daily for 30 days beginning 24 h after ischemic stroke. No significant differences were observed between the resveratrol and placebo groups regarding blood pressure, NIHSS score, Barthel Index, or Modified Rankin Scale, suggesting that short-term resveratrol monotherapy may not improve functional recovery after stroke [104]. Findings from clinical trials assessing the effects of resveratrol on stroke treatment, post-stroke disability, and stroke-related risk factors are summarized in Table 3.

### 5.6. Data on Resveratrol’s Cardiological and Neurological Safety, and Interactions with Stroke Pharmacotherapy

#### 5.6.1. Resveratrol’s Adverse Effects

Resveratrol is generally considered to be well tolerated when administered to healthy individuals at commonly used supplemental doses. However, in vitro and in vivo studies have demonstrated that, once a certain concentration threshold is exceeded, its pharmacological activity may shift towards pro-oxidant and potentially cytotoxic effects. This dose-dependent response is essential for interpreting the available data on its cardiovascular and neurological safety [105]. Moreover, excessive resveratrol intake may result in clinically relevant interactions with multiple medications, particularly in patients receiving polypharmacy. Clinical studies generally indicate favourable tolerability, with adverse effects being predominantly dose-dependent and usually limited to gastrointestinal symptoms, including nausea, abdominal discomfort, and diarrhoea, as well as headache, dizziness, and fatigue. Cognitive assessments conducted at moderate and high doses have shown no evidence of adverse cognitive effects. A dose of 300 mg/day was not associated with cognitive impairment, while an isolated improvement in psychomotor speed was observed at the higher dose of 1000 mg/day. To date, no serious or irreversible neurotoxicity has been reported [106]. In a rodent study conducted by Crowell et al., a very high dose of resveratrol (3000 mg/kg) caused fatal cardiac and renal toxicity. At a lower dose of 300 mg/kg, pure resveratrol exerted cardioprotective effects. However, it became cardiotoxic at the highest doses tested, whereas a modified formulation maintained its protective effects across the same dose range [106,107]. In human clinical studies, higher supplemental doses exceeding 1 g/day have been associated with increased levels of cardiovascular risk markers, including oxLDL and soluble markers of endothelial adhesion and fibrinolysis. These effects were not observed at lower doses. In addition, elevated concentrations of resveratrol may increase intracellular oxidative stress in human endothelial cells, leading to mitochondrial depolarisation and endothelial cell death. Taken together, the available evidence suggests that the cardiovascular toxicity of resveratrol is primarily associated with high-dose exposure [105,106].

#### 5.6.2. Resveratrol’s Interactions with Drugs Related to Stroke Treatment

The clinically relevant interaction potential of resveratrol arises primarily from its effects on cytochrome P450 enzymes and the intestinal efflux transporter P-glycoprotein. These effects are further influenced by extensive presystemic conjugation in the intestine and liver, as well as by high plasma protein binding, which together determine the proportion of free resveratrol available to interact with co-administered medications [105]. Among the cytochrome P450 isoforms investigated, resveratrol exhibits its most potent inhibitory activity against CYP3A4. Studies have demonstrated time- and NADPH-dependent irreversible inactivation of this enzyme, with kinetics comparable to those of bergamottin, the compound largely responsible for the grapefruit juice drug interaction. In healthy volunteers, daily resveratrol supplementation increased exposure to a poorly bioavailable CYP3A4 substrate, consistent with reduced intestinal first-pass metabolism. Animal pharmacokinetic studies involving two CYP3A4-metabolised calcium channel blockers similarly demonstrated increased oral exposure. Together with the in vitro inhibition of P-glycoprotein observed at higher concentrations, these findings suggest that resveratrol may enhance intestinal drug absorption through combined CYP3A4- and transporter-mediated mechanisms, rather than by altering hepatic clearance.

This interaction is clinically relevant to several classes of drugs commonly used in cardiovascular and post-stroke care that undergo substantial CYP3A4-dependent first-pass metabolism. These include statins such as atorvastatin, lovastatin, and simvastatin; dihydropyridine and other calcium channel blockers, including felodipine, nicardipine, nifedipine, nisoldipine, nitrendipine, nimodipine, and verapamil; and the antiarrhythmic agent amiodarone. For these medications, high-dose resveratrol supplementation may increase systemic exposure and the risk of dose-related toxicity, particularly in the case of drugs with a narrow therapeutic index [105,106,108].

Several studies have also shown that resveratrol can alter CYP2C9 activity, which is a major metabolic pathway for warfarin. This pharmacokinetic interaction is supported by pharmacodynamic evidence indicating that resveratrol may enhance the anticoagulant effect of warfarin and thereby increase the risk of bleeding [108]. In addition, the antiplatelet effects of resveratrol observed in post-infarction patients raise further concerns regarding bleeding when it is co-administered with warfarin and, potentially, with other agents that affect haemostasis. However, no relevant evidence has yet been identified regarding interactions with non-warfarin anticoagulants, antiplatelet agents, or thrombolytic drugs [105,106,108].

Overall, the available evidence suggests that resveratrol consumed at low, diet-equivalent doses carries a minimal risk of clinically significant drug interactions. In contrast, higher supplemental doses warrant caution in patients receiving calcium channel blockers, warfarin, or other drugs with a narrow therapeutic index that depend on CYP3A4- or CYP2C9-mediated metabolism.

## 6. Curcumin in Stroke-Related Neuroprotection: Experimental and Clinical Evidence

### 6.1. Evidence from In Vitro Studies

**Figure 3:**
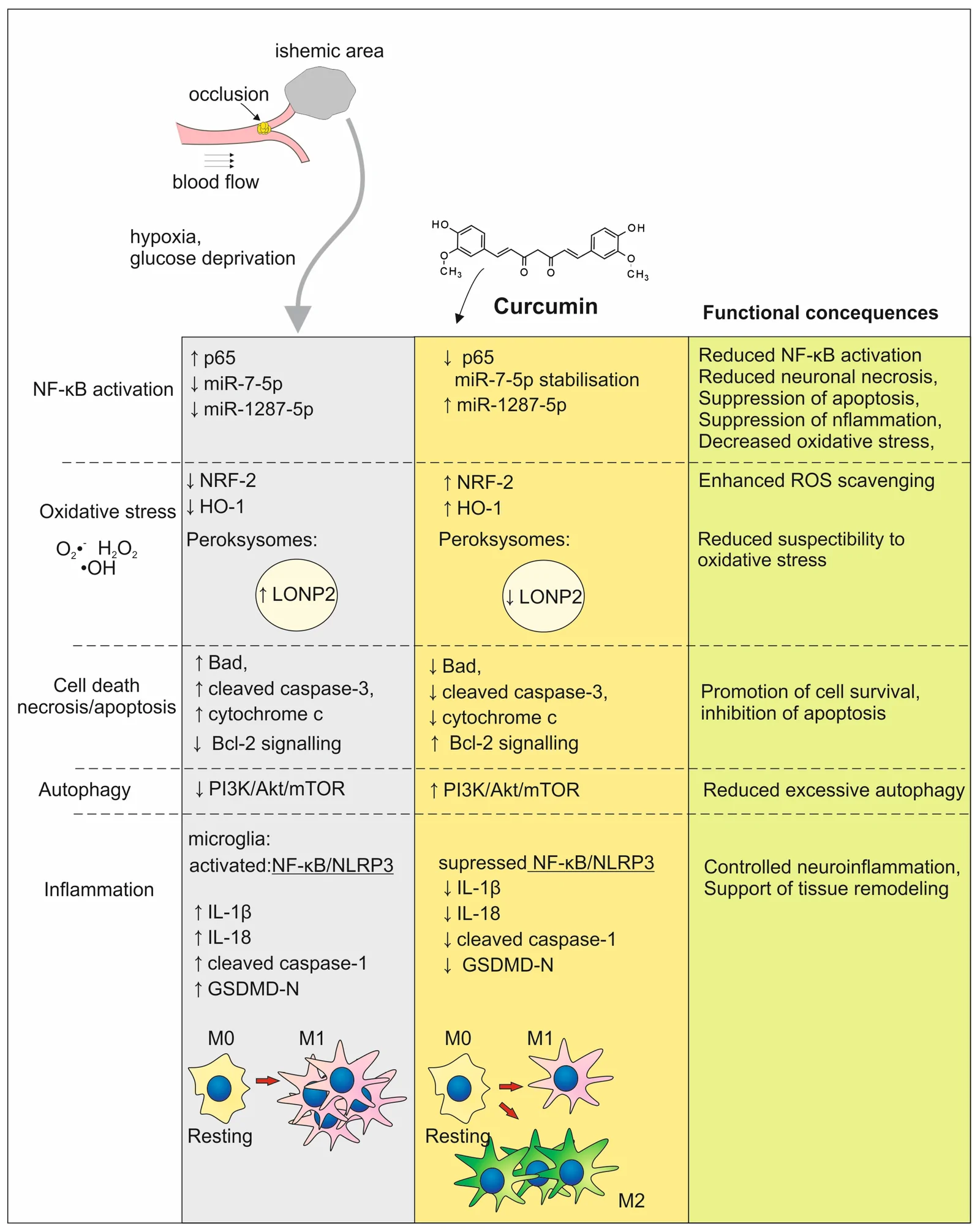
Summary of the principal molecular alterations induced by cerebral ischemia and their modulation by curcumin. Cerebral ischemia promotes NF-κB activation, oxidative stress, apoptosis, dysregulated autophagy and neuroinflammation, leading to neuronal injury. Curcumin counteracts these pathological processes by suppressing NF-κB signalling, modulating miR-7-5p and miR-1287-5p expression, activating the NRF2/HO-1 antioxidant pathway, reducing LONP2 expression, restoring PI3K/Akt/mTOR signalling, inhibiting NLRP3 inflammasome activation and pyroptosis, and promoting microglial polarization toward the anti-inflammatory M2 phenotype. Collectively, these effects reduce oxidative stress, apoptosis, excessive autophagy and neuroinflammation, thereby improving neuronal survival and supporting tissue repair after stroke. Notation: ↑ means upregulation/increase; ↓ means downregulation/decrease.

Neuronal damage induced by oxidative stress is a key factor in the pathogenesis of injury associated with ischaemia and subsequent reperfusion. Conditions resembling this state can be reproduced in vitro by depriving cell cultures of nutrients and oxygen. Xu et al. demonstrated that PC12 cells treated with curcumin in an OGD/R model preserve microRNA-7-5p expression while simultaneously exhibiting reduced p65 levels. These alterations lead to suppression of necrosis, apoptosis, inflammation, and oxidative stress, thereby mitigating the mechanisms responsible for brain injury and cognitive dysfunction following cerebral ischaemia [109]. Another study using the same model in SH-SY5Y cells showed that curcumin also reverses OGD/R-induced downregulation of microRNA-1287-5p and upregulation of lon peptidase 2, peroxisomal (LONP2). Overexpression of microRNA-1287-5p was found to reduce ROS-induced injury. Conversely, activation of LONP2 exacerbated neuronal damage. Therefore, its downregulation by curcumin further enhanced the protective effect. The authors also highlighted curcumin’s ability to inhibit apoptosis and increase mitochondrial membrane potential in neuroblastoma cell cultures. Repression of apoptosis was associated with inhibition of pro-apoptotic factors, including Bcl-2-associated agonist of cell death, cleaved caspase-3, and cytochrome c, together with enhancement of anti-apoptotic mechanisms mediated by Bcl-2 [110,111]. Xie et al. reported consistent findings regarding modulation of mitochondrial membrane potential and the expression profiles of apoptosis-related proteins in an N2A cell line [112]. Curcumin also provides substantial neuroprotection in primary microglial cultures exposed to pyroptosis-promoting conditions. Culture medium supplemented with curcumin significantly reduces the levels of pyroptosis-related proteins, including the N-terminal fragment of gasdermin D, cleaved caspase-1, IL-1β, and IL-18, as well as suppresses activation of the NF-κB/NLRP3 inflammasome pathway [113]. Figure 3 summarizes the major molecular alterations induced by cerebral ischemia and their modulation by curcumin, highlighting the resulting neuroprotective effects.

### 6.2. Evidence from Animal Models

#### 6.2.1. The Administration of Curcumin After the Onset of Ischemia

Multiple animal studies have documented a broad preclinical neuroprotective profile of curcumin, including anti-inflammatory, antioxidant, and anti-apoptotic effects in experimental stroke models. Oral administration of curcumin significantly improves neurological outcomes in diabetic rats following MCAO surgery. Post-mortem analyses of these animals have shown that administration of this phytochemical reduces both infarct size and brain oedema volume. Stabilization of glucose transporter 1 and glucose transporter 3 (GLUT-1 and GLUT-3) expression, as well as anti-apoptotic modulation of Bax and Bcl-2 levels, are considered essential mechanisms underlying the amelioration of this condition [114]. Other studies using similar experimental models have shown that curcumin also reduces the production of inflammatory cytokines such as IL-1, IL-6, and TNF-α, alleviates mitochondrial dysfunction in ischaemic brain tissue by counteracting ROS generation, and promotes M2 microglial polarization. This functional microglial phenotype is responsible for tissue repair following ischaemia-induced injury. Moreover, curcumin alters the expression patterns of p53 and SIRT1, which further contributes to inhibition of apoptosis [115,116,117]. An additional explanation for the neuroprotective effects of curcumin may lie in its ability to promote neurogenesis through RNA regulatory networks, including lncRNA, circRNA, miRNA, mRNA and ceRNA interactions [118]. Shah et al. also pointed out that administration of curcumin after the onset of cerebral ischaemia positively affects the activity of molecular components involved in maintenance of cellular homeostasis, particularly ubiquitin carboxyl-terminal hydrolase L1 (UCH-L1), isocitrate dehydrogenase (IDH), adenosylhomocysteinase (SAH), and eukaryotic initiation factor 4A (eIF4A) [119]. Available evidence regarding autophagy in stroke indicates that its role depends on the stage of the disease. Although this process may be protective during the early phases of ischaemia, it may also exacerbate damage during reperfusion [120]. Administration of curcumin shortly after cerebral artery occlusion enhances neurological recovery and inhibits excessive autophagy via the PI3K/Akt/mTOR pathway. In addition, signalling through the toll-like receptor 4 (TLR4)/p38/mitogen-activated protein kinase (MAPK) pathway is also reduced [117]. The effects of treatment with this polyphenol are also evident at the level of individual transcription factors. Curcumin stimulates NRF2, which enhances multiple antioxidant pathways through its downstream effector HO-1. This mechanism contributes to protection against lipid peroxidation, as reflected by lower MDA formation. In turn, inhibition of NF-κB is associated with suppression of the excessive inflammatory response, which would otherwise further exacerbate ischaemic injury [121,122]. The timing and dose of curcumin administration are pivotal in determining its therapeutic efficacy. The earlier curcumin is administered after the onset of ischaemia, the better the potential outcome. Likewise, increasing doses are associated with broader beneficial effects. In several rodent MCAO studies, a dose of 300 mg/kg has yielded the most favorable experimental endpoints. This optimal dose consistently results in the greatest reductions in infarct size, brain edema volume, and neurological (motor and cognitive) deficits. At the cellular level, 300 mg/kg is highly effective at counteracting NO and peroxynitrite production, thereby mitigating oxidative stress. Furthermore, it prevents excessive apoptosis of hypoxic neurons by significantly limiting the release of cytochrome c and cleaved caspase-3. However, these findings should not be interpreted as directly translatable to humans without dedicated pharmacokinetics and safety assessment [123,124,125].

#### 6.2.2. The Administration of Curcumin Prior to the Onset of Ischemia

In a study by Lan et al., it was demonstrated that the protective effect of curcumin pretreatment against stroke observed in SHRSP models is not a consequence of blood pressure (BP) reduction, but rather of alleviation of oxidative stress through the UCP2/nitric oxide (NO) pathway. Administration of curcumin to hypertensive rats does not result in significant changes in BP, body weight, or brain-to-body weight ratio. However, it delays stroke onset and prolongs survival. Moreover, this phytochemical increases carotid artery relaxation in a dose-dependent manner, enhancing responsiveness to acetylcholine and sodium nitroprusside. It also increases plasma SOD activity and NO levels, while simultaneously reducing MDA and ROS levels in the basilar artery wall [126]. Pretreatment with curcumin significantly reduces the expression of pro-inflammatory cytokines in albino rats subjected to spinal cord ischaemia–reperfusion injury [127]. Moreover, de Alcântara et al. demonstrated that such prophylactic intervention enhances neuronal viability and attenuates the immunoreactivity of TNF-α and cyclooxygenase-2 (COX-2) in a rat model of cerebral ischaemic injury. Curcumin has also been shown to modulate the balance of individual neurotransmitter systems. Ischaemia-induced depletion of dopamine and its metabolites, 3,4-dihydroxyphenylacetic acid and homovanillic acid, can be reversed by curcumin treatment. A similar effect has been observed for norepinephrine and serotonin levels [128]. Pretreatment with curcumin also appears to affect mitochondria in rats subjected to MCAO, contributing to preservation of mitochondrial membrane potential and upregulation of mitochondrial complex I activity. Together with the reduction in cytoplasmic cytochrome c levels, stimulation of SIRT1, promotion of p53 deacetylation, and stabilisation of Bcl-2 and Bax expression, curcumin exerts a multifaceted anti-apoptotic effect [129].

#### 6.2.3. Long-Term Curcumin Supplementation

As discussed above, curcumin exerts a promising protective effect in animal models of stroke, both when administered as pretreatment and after the onset of cerebral ischaemia. Studies in which curcumin was administered before cerebral artery occlusion and continued after injury provide additional preclinical support for its neuroprotective profile. Rats subjected to a 5-day course of curcumin pretreatment followed by 3 days of post-MCAO curcumin administration show significant improvement in neurobehavioural outcomes, along with reductions in infarct volume and overall mortality. At the histological level, these effects are reflected in reduced lipid peroxidation and lower intracellular calcium accumulation [130]. Liu et al. postulated that one of the signalling pathways involved in mediating these aspects of neurofunctional recovery is the Notch pathway, as evidenced by increased levels of the Notch intracellular domain [131]. Further studies investigating even longer periods of curcumin administration have shown that such chronic supplementation favourably modulates intercellular inflammatory signalling, as reflected by reduced secretion of IL-6 and TNF-α. In addition, the overall redox balance is positively affected, with increased activity of SOD, GPx, and CAT, as well as decreased MDA levels [132]. A possible explanation for the accompanying attenuation of cognitive impairment induced by bilateral common carotid artery occlusion (BCCAO) and four-vessel occlusion-induced brain ischaemia (4VOI) may be the promotion of neurogenesis together with inhibition of excessive apoptosis. Prolonged curcumin administration enhances neural stem cell proliferation and their subsequent differentiation into mature neurons within the hippocampus. This effect is associated with increased expression of neurogenin 2 (Ngn2), paired box 6 (Pax6), and neurogenic differentiation 1 (NeuroD1), as well as enhancement of Wnt/β-catenin signalling [133]. Furthermore, improved acetylcholine transmission can also be observed, which may additionally contribute to better cognitive function [134]. Results from in vivo studies regarding curcumin’s role in treatment of stroke are summarized in Table 4.

### 6.3. Curcumin Formulations and Structural Modifications Designed to Improve Bioavailability and Target-Tissue Delivery

Although unmodified curcumin shows notable anti-inflammatory and antioxidant properties, its clinical translation is constrained by poor oral absorption, low water solubility, chemical instability, and low bioavailability [88,135,136]. Most researchers emphasise the potential of curcumin nanoformulations for effective brain targeting and for crossing the BBB. Curcumin nanoemulsions have been shown to promote behavioural recovery, reduce haematoma size, and mitigate weight loss more effectively than native curcumin preparations in rats following ICH [137]. At the same time, curcumin nanoparticles (CNPs) have shown promising effects in alleviating ischaemic damage in brain tissue. Their administration significantly improves motor function, muscle strength, and the activity of antioxidant enzymes, including GPX, glutathione S-transferase (GST), SOD, and CAT, thereby reducing oxidative stress [136]. Another strategy for improving the pharmacological properties of polyphenols involves the use of exosomes. These vesicular structures can serve as carrier particles and allow incorporation of various peptides into their membrane, where they act as targeting ligands for specific receptors. This, in turn, facilitates uptake by particular cell types, making the intervention more selective for damaged regions and tissues. The cyclo(Arg-Gly-Asp-D-Tyr-Lys) peptide (cRGDyK) may serve as such a molecular anchor because of its high affinity for integrin αvβ3, which is specifically expressed in reactive cerebral vascular endothelial cells after ischaemia. In the MCAO model, exosomes containing cRGDyK significantly enhance the beneficial effects of curcumin by providing more potent suppression of both inflammation and apoptosis. Moreover, their application does not appear to produce discernible toxicity or adverse effects [135]. Zhang et al. introduced BDP-4/Cur-CL nanoparticles as a novel approach to address the critical challenges of early detection and timely intervention in ischaemic stroke. These particles are created by combining aggregate-induced emission luminogens with modified curcumin molecules. After activation by endogenous peroxynitrite (ONOO−), they emit near-infrared afterglow signals, enabling real-time localisation of ischaemia–reperfusion injury. At the same time, they are capable of releasing curcumin selectively within stroke-affected areas, thereby improving the therapeutic effect. Research performed in mice subjected to MCAO showed that these complex formulations counteract apoptosis, reduce neuroinflammation, minimise brain tissue damage, and improve cognitive function more effectively than conventional CNPs [138]. An alternative strategy involving composite particles uses polysaccharides derived from the root of Angelica sinensis modified with sialic acid residues. These carriers allow conjugated curcumin to cross the BBB more effectively, thereby providing superior neuronal protection against expansion of ischaemic lesions [88]. Modifications of the chemical structure of curcumin itself, without the use of carrier particles, may also enhance its therapeutic potential. In a rabbit small clot embolic stroke model, administration of a pyrazole derivative of curcumin, designated CNB-001, resulted in better functional outcomes with respect to neurological impairment, which was attributed to its greater ability to penetrate the BBB. CNB-001 also demonstrated the ability to preserve signalling pathways involving key protein kinases. This was particularly evident in maintenance of extracellular signal-regulated kinase (ERK) and Akt phosphorylation, as well as stabilisation of mitogen-activated protein kinase and PI3K-Akt pathway activity. Moreover, it preserved proper Ca2+/calmodulin-dependent protein kinase II alpha (CaMKIIα) activity, which is critical for neuronal function, and maintained the activity of the pro-survival chaperone 150 kDa oxygen-regulated protein (ORP150) [139]. Further studies using the same compound in rabbit models of cerebral ischaemia expanded these findings by showing that CNB-001 increases BDNF levels, thereby supporting synaptic plasticity. In addition, it was shown to inhibit 5-lipoxygenase and 15-lipoxygenase, as well as COX-2, resulting in marked suppression of inflammatory processes [140]. Reports from studies assessing bioavailability and effectiveness of curcumin particles, modified with chemical alterations or carrier compounds, are summarized in Table 5.

### 6.4. The Enhancing Potential of Curcumin on Other Therapy Forms

Preclinical findings suggest that curcumin may warrant evaluation as an adjunctive candidate in stroke-related neuroprotection. Experimental studies also suggest that curcumin may augment selected recovery-related endpoints when combined with other investigational or standard therapeutic platforms, although this has not been clinically validated. One study demonstrated that curcumin improves the survival of human umbilical cord-derived mesenchymal stem cells (hUC-MSCs) via the ERK1/2 pathway. Transplantation of these cells represents one of the non-conventional approaches aimed at improving outcomes in patients and experimental animals after cerebral ischaemia [141]. In addition to improving cell viability, curcumin also increases the therapeutic value of hUC-MSC transplantation. Analyses performed in rodents subjected to MCAO revealed that this combined therapy produces the most favourable outcomes in terms of infarct size reduction, attenuation of cerebral oedema, alleviation of neurological deficits, and stimulation of anti-inflammatory and antioxidant responses via the Akt/glycogen synthase kinase 3β/β -transducin repeat-containing protein/NRF2 axis [142]. Another investigational cell-based approach being explored in animal models of cerebral ischaemia involves olfactory mucosa mesenchymal stem cells (OM-MSCs). Addition of curcumin enhances their efficacy in the treatment of ICH in rats. Combined therapy results in more effective inhibition of ferroptotic neuronal death and greater antioxidant capacity than either curcumin alone or cell transplantation alone [143]. Similarly, Lan et al. investigated the effects of curcumin-preconditioned OM-MSCs in cerebral ischaemia–reperfusion injury rather than ICH. This study provided the first evidence of panoptosis in this context, demonstrating significant upregulation of markers associated with pyroptosis, apoptosis, and necroptosis in both HT22 cells exposed to OGD/R and mice subjected to cerebral artery occlusion. Curcumin-preconditioned OM-MSCs attenuate panoptotic neuronal death by modulating microglial polarisation through the nucleotide-binding oligomerization domain-containing protein 2/NF-κB/MAPK pathway and microRNA-423-5p, thereby promoting the anti-inflammatory M2 microglial phenotype [144]. One of the major risk factors for both ischaemic and haemorrhagic stroke is hypertension, which increases the likelihood of cerebral vessel injury, blood extravasation into surrounding tissues, and intravascular thrombosis. Among antihypertensive drugs, angiotensin II receptor blockers are commonly used and are particularly recommended for individuals with a history of stroke. In a mouse MCAO model, combined treatment with curcumin and the ARB candesartan resulted in enhanced cerebral blood flow, increased SOD and GST activity, stabilisation of heart rate, and a reduction in oxidative stress markers compared with untreated controls and animals treated with either curcumin or the ARB alone [145].

### 6.5. Evidence from Clinical Trials

Boshagh et al. conducted a pioneering clinical trial to investigate the effects of a curcumin-piperine complex on clinical and biochemical parameters in patients with stroke. Piperine was included to enhance the pharmacokinetic properties of curcumin. A total of 66 participants were randomly assigned to receive either curcumin-piperine tablets (500 mg curcumin + 5 mg piperine) or placebo for 12 weeks. The curcumin–piperine group showed improvements in carotid intima–media thickness and BP values. Reductions were also observed in CRP, total cholesterol, triglycerides, body weight, and waist circumference, together with an increase in antioxidant capacity. Only minimal adverse effects were reported. This trial provides preliminary clinical support for favourable effects on vascular, metabolic, inflammatory, and oxidative stress-related markers during the rehabilitation phase. However, it does not establish curcumin as an effective treatment for acute stroke or as a proven strategy for preventing recurrent stroke [146]. Most of the remaining clinical evidence is indirect and derives from non-stroke populations. In a randomized, double-blind, placebo-controlled trial, healthy older adults receiving a highly bioavailable curcumin formulation (curcuRouge™, 90 mg twice daily) for 4 weeks showed a significant reduction in the neutrophil-to-lymphocyte ratio (NLR), accompanied by decreased neutrophil counts and increased lymphocyte proportions, without any reported adverse events, supporting the anti-inflammatory potential of curcumin [147]. Because elevated NLR is associated with poor functional outcomes after ischemic stroke, these findings may have clinical relevance, although they require confirmation in stroke patients [147,148]. Similarly, supplementation with a combined nutraceutical containing curcumin, eicosapentaenoic acid, astaxanthin, and γ-linolenic acid for 4 weeks significantly reduced systolic BP, improved endothelial function, and attenuated inflammatory markers (specifically CRP and IL-6) in healthy adults. Because the intervention contained several active compounds, these effects cannot be attributed specifically to curcumin. However, they support the concept that modulation of inflammation and endothelial dysfunction may reduce stroke-related cardiovascular risk factors [149]. In contrast, a randomized, double-blind, placebo-controlled trial involving 152 overweight and obese older adults found that 160 mg/day of optimized curcumin (Longvida^®^) administered for 16 weeks, either alone or combined with fish oil, did not improve cerebrovascular responsiveness, cerebral blood flow, arterial stiffness, inflammatory markers, BP, lipid profile, or other cardiometabolic parameters compared with placebo [150]. Overall, current clinical evidence suggests that curcumin may favorably influence selected inflammatory, oxidative, vascular, and metabolic markers relevant to stroke recovery and risk reduction. However, these effects appear to depend partly on the formulation and co-administered compounds, remain inconsistent across studies, and are insufficient to confirm efficacy in acute stroke treatment or secondary prevention.

### 6.6. Data on Curcumin’s Cardiological and Neurological Safety, and Interactions with Stroke Pharmacotherapy

#### 6.6.1. Curcumin’s Adverse Effects

Across multiple studies, curcumin has demonstrated a consistently favourable safety profile. Reported adverse effects are predominantly gastrointestinal and include dyspepsia, abdominal pain, nausea, and diarrhoea. No serious cardiac or neurological adverse events have been reported, even at high oral doses of 4000 and 8000 mg/day. Mild neurological adverse effects, such as headache, dizziness, and fatigue, appear to be infrequent [151,152]. In randomized trials specifically designed to assess potential cardiac toxicity, curcumin administered at a dose of 80 mg/day for five days did not affect ejection fraction, left ventricular function, or the incidence of arrhythmias in patients with unstable angina. Similarly, administration of nanomicellar curcumin before elective percutaneous coronary intervention did not increase troponin I or creatine kinase-MB levels, providing no evidence of clinically relevant cardiotoxicity [153]. An important factor contributing to the favorable safety profile of curcumin is its inherently poor oral bioavailability, which limits systemic exposure and may consequently reduce the risk of dose-dependent toxicity. However, this protective effect may be less pronounced when bioavailability-enhanced formulations are used, as these preparations can produce substantially higher systemic concentrations [154,155].

#### 6.6.2. Curcumin’s Interactions with Drugs Related to Stroke Treatment

Human clinical studies of curcumin-drug interactions have generally been limited to relatively low doses of curcumin, typically 200–300 mg/day. By contrast, most available evidence derives from single-dose or short-term animal studies using doses substantially higher than those commonly consumed as supplements, often exceeding 60–100 mg/kg/day. Consequently, the clinical relevance of long-term or high-dose interactions, as well as their potential implications for therapeutic efficacy and toxicity, requires further investigation [153,155,156,157]. The drug interaction potential of curcumin is primarily pharmacokinetic and is associated with the inhibition of several cytochrome P450 isoenzymes, including CYP1A2, CYP2B6, CYP2C9, CYP2C19, CYP2D6, and CYP3A4, as well as the efflux transporter P-glycoprotein (P-gp). Because many substrates of P-gp and CYP enzymes are drugs with a narrow therapeutic index, curcumin-induced increases in their plasma concentrations could potentially exacerbate adverse effects or toxicity. However, the available evidence is inconsistent regarding the magnitude and clinical relevance of these interactions [155,156].

Curcumin does not appear to prolong bleeding time when co-administered with aspirin or ticlopidine [157]. However, it may alter the plasma concentrations of antiplatelet agents such as clopidogrel, which is partially converted by CYP2C19 and CYP3A4 into its active platelet-inhibiting metabolite. Nevertheless, because this CYP-dependent activation pathway appears to make only a limited contribution to the overall pharmacological effect, curcumin has not been shown to produce a clinically meaningful change in platelet inhibition or bleeding time [156].

The effects of curcumin on direct oral anticoagulants used in post-stroke care remain uncertain. Hu et al. reported that co-administration of curcumin with dabigatran did not significantly alter INR, despite dabigatran being a P-gp substrate that is not metabolised by cytochrome P450 enzymes [157]. In contrast, rivaroxaban, apixaban, and edoxaban depend to varying degrees on both CYP3A4 and P-gp pathways. This raises the possibility that inhibitors of these pathways, including curcumin, could increase systemic drug exposure and bleeding risk, although further clinical research is required to determine the significance of this interaction [158].

Warfarin may be particularly susceptible to interactions with curcumin because of its narrow therapeutic index and its dependence on CYP2C9, as well as CYP1A2, CYP2C19, and CYP3A4, for metabolism [158]. In a study by Liu et al., high-dose curcumin increased the maximum plasma concentration of warfarin in rats. However, the elimination half-life of warfarin remained unchanged, suggesting that the effect was more likely attributable to enhanced absorption caused by P-gp inhibition than to altered CYP2C9-mediated metabolism. The increase in maximum plasma concentration did not result in a significant change in prothrombin time. Nevertheless, the authors emphasised that the clinical significance of this interaction remains unclear and cautioned that high-dose curcumin combined with warfarin could potentially enhance anticoagulant activity and lead to unexpected bleeding [156]. In addition, Hu et al. reported that co-administration of a curcumin–phosphatidylcholine complex with warfarin or dabigatran did not alter bleeding time in patients receiving chronic antiplatelet therapy [157].

Beyond anticoagulant and antiplatelet therapy, curcumin may modestly alter the plasma concentrations of antihypertensive agents such as celiprolol, talinolol, nifedipine, and valsartan. However, the long-term consequences of repeated co-administration for efficacy and safety remain unconfirmed. Because several of these drugs are substrates or inhibitors of CYP3A4 and P-gp, negative findings from single-dose studies should not be regarded as definitive evidence of long-term safety. Such findings do not exclude clinically relevant interactions that could affect blood-pressure control or increase toxicity [153,155].

Among lipid-lowering agents, curcumin has been shown to increase rosuvastatin exposure in rats and dogs, potentially through inhibition of hepatic organic anion-transporting polypeptides OATP1B1 and OATP1B3 by curcumin conjugates. Because statin-associated myopathy is a dose-dependent adverse effect, increased systemic exposure could theoretically elevate the risk of myopathy or other statin-related toxicities. However, no corresponding clinical adverse events have yet been reported for this specific combination [155].

Overall, the pharmacokinetic interactions of curcumin vary across drug classes. At commonly used supplemental doses, curcumin appears unlikely to produce acute cardiovascular or neurological harm. Nevertheless, because it can measurably affect the absorption, metabolism, or transport of drugs that are substrates of P-gp, CYP3A4, CYP2C9, or OATP transporters, it may either reduce therapeutic efficacy or increase toxicity, depending on the affected drug and the direction of the interaction. Patients receiving anticoagulants, antiplatelet agents, or antihypertensive medications, particularly those with a narrow therapeutic index, may therefore require closer clinical monitoring when using curcumin supplements [155,156].

## 7. Gut Microbiome and Stroke Risk-Modulatory Effects of Resveratrol and Curcumin

### 7.1. The Gut Microbiome as a Modulator of Stroke Risk and Treatment Response

The gut microbiome is recognised as an important component of the gut–brain axis that may influence both stroke susceptibility and the response to cerebral ischemia. This relationship is bidirectional: intestinal dysbiosis may contribute to vascular risk, whereas acute stroke can alter gut motility, intestinal permeability, immune function, nutrition, and microbial composition [159,160,161]. Consequently, microbiome changes observed after stroke may represent both pre-existing risk factors and secondary consequences of brain injury. Patients with ischemic stroke frequently show reduced abundance of short-chain fatty acid-producing bacteria, including *Faecalibacterium*, *Roseburia*, and members of Lachnospiraceae, together with an increased abundance of potentially pro-inflammatory taxa such as the Enterobacteriaceae group [159,160,161]. A higher Stroke Dysbiosis Index has been associated with greater neurological impairment and poorer functional outcome. Moreover, transfer of microbiota from patients with marked dysbiosis to experimental animals increased ischemic brain injury and promoted intestinal interleukin-17-producing γδ T-cell responses, suggesting that microbial alterations may actively modulate post-stroke inflammation [160]. Microbial metabolites appear to be particularly important in this interaction. Short-chain fatty acids, especially acetate, propionate, and butyrate, support intestinal barrier integrity and regulate immune responses, endothelial function, and microglial activity. Reduced concentrations of these metabolites have been associated with greater stroke severity and worse 90-day functional outcomes [161]. Their deficiency may facilitate systemic inflammation and neuroinflammation by weakening the intestinal barrier and altering immune-cell differentiation. In contrast, some microbiota-derived metabolites may increase vascular and thrombotic risk. Trimethylamine N-oxide (TMAO), produced from dietary choline and L-carnitine through bacterial and hepatic metabolism, enhances platelet reactivity and thrombus formation [162]. Higher circulating TMAO concentrations have been associated with incident ischemic stroke, although this relationship is influenced by diet, renal function, age, and cardiometabolic disease [163]. Another microbial co-metabolite, phenylacetylglutamine, can enhance platelet activation through adrenergic receptors, providing an additional mechanism linking microbial metabolism with thrombosis [164]. However, neither metabolite has yet been established as a clinically useful biomarker or therapeutic target in stroke. Dysbiosis may also worsen stroke outcome by increasing intestinal permeability. Disruption of epithelial tight junctions facilitates the translocation of bacterial components, including lipopolysaccharide, into the circulation, thereby promoting systemic inflammation, endothelial dysfunction, and immunothrombosis. At the same time, stroke-induced immunosuppression may permit dissemination of intestinal bacteria and increase susceptibility to post-stroke infections [165]. The gut microbiome may additionally contribute to interindividual variation in the efficacy and safety of drugs used in stroke prevention [166]. Baseline microbiota composition has been associated with differences in statin response, whereas statin treatment itself may reduce pro-inflammatory microbial patterns [167]. Intestinal bacteria may also influence warfarin sensitivity through microbial vitamin K production. Enrichment of *Escherichia–Shigella* and increased vitamin K_2_ biosynthetic capacity have been associated with reduced anticoagulant response [168]. Preclinical findings further indicate that microbial metabolism may modify aspirin exposure and the effects of selected antihypertensive agents [169,170]. Nevertheless, there is currently insufficient evidence to use microbiome profiles to guide antiplatelet, anticoagulant, thrombolytic, or antihypertensive therapy in patients with stroke. Overall, available evidence supports a plausible relationship between microbial composition, microbial metabolites, vascular risk, post-ischemic inflammation, and pharmacological response [171]. However, most human studies remain observational, and their results are affected by diet, comorbidities, medication use, geography, and sampling time. These mechanisms nevertheless provide a rationale for investigating polyphenols such as resveratrol and curcumin. In addition to their direct anti-inflammatory, antioxidant, endothelial, and antiplatelet effects, these compounds may influence stroke-related pathways by modifying microbial composition, short-chain fatty acid production, intestinal barrier integrity, and the generation of proatherogenic metabolites [171,172].

### 7.2. Potential Mechanisms Linking Resveratrol and the Gut Microbiota Modulation in Stroke Prevention and Treatment

Interest in resveratrol as a cardio- and cerebrovascular agent has long been constrained by a pharmacokinetic paradox: although more than 70% of an oral dose is absorbed, only about 1–8% of circulating resveratrol remains in its free form. The remainder is rapidly conjugated into glucuronides and sulfates, resulting in plasma concentrations of the parent compound that are far lower than those of its metabolites [173]. This has led to the hypothesis that poorly bioavailable phenolic phytochemicals act primarily within the gut rather than systemically. This hypothesis is supported by the observation that caecal resveratrol concentrations in treated mice greatly exceed plasma concentrations [174]. Unabsorbed resveratrol is extensively biotransformed by the colonic microbiota into dihydroresveratrol, lunularin and 3,4′-dihydroxy-trans-stilbene [173]. Dihydroresveratrol inhibits NO synthesis to an extent comparable to that of the parent compound, whereas 3,4′-dihydroxy-trans-stilbene is more effective than resveratrol in counteracting insulin resistance [173]. Metabolite profiles differ markedly among individuals, and microbiota composition may determine whether supplementation produces a biologically meaningful effect. In the study by Bird et al., resveratrol exhibited broad but selective antibacterial activity, inhibiting most of a panel of 43 bacterial strains while sparing or even stimulating commensals such as *Lactobacillus acidophilus* [173]. In rodents, the most consistently reported alteration in microbiota composition is an increase in the *Bacteroidetes*/*Firmicutes* ratio. Resveratrol also increased the abundance of *Bacteroides, Lactobacillus, Bifidobacterium*, and *Akkermansia*, while reducing *Prevotella*, Ruminococcaceae, *Anaerotruncus*, *Alistipes*, *Helicobacter*, and Peptococcaceae [173,174]. Notably, in several models, resveratrol affected the microbiome only in animals fed an obesogenic diet, suggesting that its action may primarily involve reversing diet-induced dysbiosis rather than disrupting a healthy microbial community [173]. Comparable effects have also been observed at dietary doses. In rats administered 1 mg/kg/day, corresponding to a human-equivalent dose of approximately 10 mg/day, resveratrol significantly increased faecal *Lactobacillus* and *Bifidobacterium* but limited the overgrowth of *Escherichia coli* and other enterobacteria following dextran sulfate sodium-induced colitis. These effects were accompanied by the preservation of mucosal architecture and reductions in colonic prostaglandin E2, COX-2, and NO. In the study by Larrosa et al., the authors proposed that the expansion of lactobacilli and bifidobacteria itself prevented colonisation and tissue invasion by enterobacteria [175]. This anti-dysbiotic effect is complemented by direct effects on epithelial integrity: resveratrol upregulates tight junction protein 2 and occludin, promotes claudin-4 assembly, and prevents the increase in intestinal permeability caused by the mycotoxin deoxynivalenol [173]. Gut barrier function is of particular relevance to stroke because experimental ischaemia itself induces dysbiosis, intestinal barrier dysfunction, and reduced motility [176]. Translocation of intestinal bacteria is a recognised source of post-stroke systemic infection; more than 70% of bacteria recovered from the lungs of patients with stroke were gut commensals such as *Enterococcus* spp., *Escherichia coli*, and *Morganella morganii*. In the study by Peh et al., mice lacking gut microbiota did not develop spontaneous post-stroke pneumonia [177]. A compound that strengthens barrier integrity and limits enterobacterial overgrowth may therefore play an important role in reducing post-stroke mortality, although this specific relationship has not yet been directly examined in clinical trials. The best-characterised microbiota-dependent pathway through which resveratrol may contribute to stroke prevention and treatment involves trimethylamine N-oxide (TMAO). TMAO is formed when gut bacteria convert dietary choline, phosphatidylcholine, and L-carnitine into trimethylamine, which is subsequently oxidised to TMAO by hepatic flavin-containing monooxygenase 3 [177]. The microbiota dependence of this process was demonstrated in a study by Tang et al., in which broad-spectrum antibiotics significantly reduced plasma TMAO levels. In a cohort of 4007 patients undergoing elective coronary angiography, those in the highest TMAO quartile had a 2.54-fold greater risk of death, myocardial infarction, or stroke over a three-year period [178]. A meta-analysis of 17 studies involving 26,167 participants confirmed a dose–response relationship between TMAO and major adverse cardio- and cerebrovascular events. The association also appears to be causal in stroke: germ-free mice receiving faecal transplants from human donors with high TMAO levels developed larger infarcts than recipients of low-TMAO microbiota, whereas choline supplementation or direct TMAO administration increased infarct volume and reduced motor activity after stroke [177]. In ApoE−/− and C57BL/6J mice, oral administration of resveratrol attenuated TMAO-induced atherosclerosis by lowering circulating trimethylamine and TMAO levels through remodelling of the gut microbiota and reduced microbial trimethylamine production from choline. These effects were not observed when resveratrol was administered parenterally [174]. A systematic review of the stroke literature likewise lists resveratrol among the dietary interventions shown to reduce TMAO levels in mice and identifies TMAO-lowering therapies as a plausible strategy for secondary prevention in at-risk patients [179]. However, evidence from human studies remains insufficient. Short-chain fatty acids (SCFAs), including acetate, propionate, and butyrate, constitute a major class of microbiota-derived metabolites. SCFAs preserve intestinal barrier integrity, suppress NF-κB-mediated inflammation, promote regulatory T-cell differentiation, and modulate microglial function after crossing the blood–brain barrier [177,180]. Their relevance to stroke has been demonstrated experimentally, as SCFA supplementation improved functional recovery, enhanced synaptic plasticity, and attenuated neuroinflammation through a gut–immune–brain axis involving circulating T cells [181]. Patients with acute ischaemic stroke exhibit significantly lower circulating SCFA concentrations, which correlate with greater stroke severity and poorer functional outcomes [177]. It remains unclear whether resveratrol significantly alters SCFA levels. Although it has consistently been shown to increase the abundance of *Lactobacillus* and *Bifidobacterium*, taxa associated with SCFA production [174,175], it also increases the *Bacteroidetes/Firmicutes* ratio, which may reduce SCFA synthesis by limiting the growth of Firmicutes [173]. An in vitro fermentation study using pharmacologically relevant concentrations of resveratrol reported reduced SCFA production alongside changes in microbial composition [173]. However, it remains unknown whether the neuroprotective effects of resveratrol are mediated by increased SCFA production. To date, no study has directly quantified SCFA concentrations following resveratrol treatment in experimental stroke models, highlighting an important gap in current knowledge. Overall, resveratrol modulates the gut microbiota through multiple pathways. In stroke prevention, resveratrol reduces microbial trimethylamine production through a TMAO-related pathway, whereas elevated TMAO levels are graded, microbiota-dependent predictors of stroke and other major cardiovascular events in humans [174,177,178,179]. Resveratrol’s ability to limit enterobacterial overgrowth and reinforce tight junctions may reduce bacterial translocation and post-stroke infection, although further research is required [173,175,177]. With regard to recovery and long-term prognosis, which depend partly on SCFA levels and T-cell-mediated modulation of microglial activation and cortical plasticity, resveratrol’s effects may remain neutral or prove ineffective if the *Bacteroidetes*/*Firmicutes* shift reduces SCFA availability [173,177,181]. Moreover, almost all available data are derived from rodent or in vitro models, frequently involving doses well above the recommended daily human intake of 450 mg, and are therefore not directly translatable across species [173]. Because resveratrol has not been administered in a stroke model in any of the studies discussed, the relationship between its microbiome-mediated effects and cerebrovascular outcomes has not been adequately investigated experimentally or clinically. Resveratrol metabolism also varies among individuals, and the microbiome itself is highly heterogeneous. In addition, across studies, 62 taxa were upregulated and 29 were downregulated after stroke [173,177]. It can therefore be concluded that resveratrol modifies both the composition of the gut microbiota and the production of microbial metabolites relevant to stroke. However, the extent to which these effects translate into stroke prevention or improved post-stroke outcomes remains uncertain.

### 7.3. Potential Mechanisms Linking Curcumin and the Gut Microbiome Modulation in Stroke Prevention and Treatment

Curcumin is characterised by low systemic bioavailability and extensive metabolism within the gastrointestinal tract. Consequently, some of its biological effects may result from local interactions with the intestinal microbiota rather than from high circulating concentrations of the parent compound. This relationship is bidirectional: curcumin can modify microbial composition and metabolism, whereas gut bacteria transform curcumin into metabolites with potentially distinct biological activities [182,183,184]. These interactions may be relevant to ischaemic stroke because intestinal dysbiosis, impaired barrier integrity, and altered microbial metabolite production have been associated with stroke risk, neuroinflammation, and functional recovery [161,181,185]. Experimental and small clinical studies indicate that curcumin can alter the abundance of several intestinal bacterial groups, including *Bacteroides*, *Clostridium*, Prevotellaceae, Bacteroidaceae, and Rikenellaceae [182,183]. However, responses vary substantially among individuals, and some participants show little or no microbiota response to supplementation [183]. This variability may depend on baseline microbial composition, diet, metabolic status, curcumin formulation, and concomitant medication use. One plausible consequence of microbiota modulation is altered production of short-chain fatty acids (SCFAs), particularly acetate, propionate, and butyrate. These metabolites support epithelial barrier integrity and influence regulatory T-cell differentiation, systemic inflammation, microglial activity, and neuronal plasticity. Reduced abundance of butyrate-producing bacteria and lower faecal SCFA concentrations have been reported in individuals at increased risk of stroke and in patients with acute ischaemic stroke. Lower SCFA concentrations have also been associated with greater neurological severity and poorer functional outcomes [161,181]. Experimental evidence suggests that SCFAs may actively support post-stroke recovery. In mice, SCFA supplementation improved motor recovery, cortical connectivity, and synaptic plasticity while modulating microglial activation through immune mechanisms involving circulating lymphocytes [181]. Curcumin could therefore influence stroke outcomes by promoting bacterial communities capable of producing SCFAs. However, no study has yet shown that curcumin increases SCFA production in an experimental stroke model or that SCFAs directly mediate its neuroprotective effects. Moreover, elevated circulating concentrations of some SCFAs have occasionally been associated with increased inflammation and poorer short-term recovery, indicating that their effects may depend on the specific metabolite, concentration, biological compartment, and stage of stroke [186]. Stroke can induce dysbiosis, reduced intestinal motility, and increased intestinal permeability. Experimental transfer of post-stroke dysbiotic microbiota has been shown to aggravate cerebral injury and promote pro-inflammatory immune responses, demonstrating that intestinal alterations can contribute to secondary brain damage [176]. Curcumin may counteract this process by protecting epithelial tight junctions. In intestinal epithelial cells, curcumin reduced lipopolysaccharide-induced inflammation and prevented disruption of proteins such as ZO-1 and claudins [187]. By limiting intestinal permeability, curcumin could reduce the translocation of lipopolysaccharide and other microbial compounds into the circulation. This may attenuate TLR4-, NF-κB-, and inflammasome-dependent signalling, thereby reducing endothelial activation, blood–brain barrier disruption, leukocyte recruitment, and neuroinflammation. This mechanism is consistent with experimental stroke studies showing that curcumin suppresses NF-κB signalling and NLRP3 inflammasome activation, decreases inflammatory cytokine production, protects white matter, and improves neurological recovery [113]. Nevertheless, these studies did not determine whether the observed effects depended on the gut microbiota. The proposed pathway linking intestinal barrier protection with reduced cerebral inflammation therefore remains plausible but unconfirmed. Curcumin may also influence microbial production of trimethylamine, the precursor of trimethylamine N-oxide (TMAO). Elevated TMAO concentrations have been associated with atherosclerosis, thrombosis, poor functional outcome, and mortality after ischaemic stroke [188]. In a clinical study of patients with non-alcoholic fatty liver disease, curcumin supplementation reduced serum trimethylamine concentrations, suggesting that it may modify microbial methylamine metabolism [189]. However, this effect has not been confirmed in patients with stroke, and it remains unknown whether curcumin-induced changes in this pathway reduce cerebrovascular risk. Another potential mechanism involves microbiota-dependent bile acid metabolism. Curcumin supplementation has been associated with changes in gut bacterial composition, secondary bile acids, TGR5 signalling, and glucagon-like peptide-1 secretion. These effects were accompanied by improvements in glucose and lipid metabolism [190]. Because diabetes, obesity, and dyslipidaemia are major stroke risk factors, modification of these signaling pathways may contribute indirectly to stroke prevention. However, no study has demonstrated that this mechanism reduces stroke incidence or improves post-stroke recovery. Gut bacteria participate directly in curcumin metabolism. Escherichia coli produces an NADPH-dependent reductase that converts curcumin into dihydrocurcumin and tetrahydrocurcumin [191]. Other microbial reactions generate additional reduced, demethylated, hydroxylated, and conjugated derivatives. Several of these metabolites retain antioxidant and anti-inflammatory activity. Studies in antibiotic-treated mice have shown that microbiota depletion substantially changes curcumin metabolism and reduces the production of specific metabolites. The microbiota may therefore determine both local intestinal exposure and the systemic availability of biologically active curcumin derivatives. Differences in curcumin-metabolising bacteria may partly explain why responses to supplementation vary among individuals [184].

## 8. Shared and Distinct Neuroprotective Mechanisms of Resveratrol and Curcumin in Ischaemic Stroke

### 8.1. Shared Neuroprotective Mechanisms of Resveratrol and Curcumin

Although resveratrol and curcumin belong to different classes of plant-derived polyphenols, based on the available evidence, it appears that the two compounds exert remarkably similar neuroprotective effects in experimental models of stroke. Rather than acting through a single molecular pathway, the phytochemicals seem to target multiple processes responsible for ischaemic brain injury in stroke, which collectively contribute to reduced infarct volume and improved neurological recovery in animal models. The principal mechanisms shared by the two phytochemicals include attenuation of inflammation and oxidative stress and inhibition of programmed cell death. Both resveratrol and curcumin suppress the activation of the NF-κB pathway and the NLRP3 inflammasome, reducing inflammatory signalling, as shown by the reduced production of cytokines including TNF-α, IL-1β, IL-6 and IL-18 [66,73,115,116,117,129]. Both compounds activate the NRF2/HO-1 antioxidant pathway, resulting in increased expression of endogenous antioxidant enzymes, including SOD, CAT and GPx [55,68,121,122]. Regulation of programmed cell death also represents a major shared mechanism, as both phytochemicals seem to inhibit excessive autophagy via the PI3K/Akt/mTOR pathway and decrease caspase-dependent apoptosis, increasing anti-apoptotic Bcl-2 expression while suppressing Bax and cleaved caspase-3, ultimately reducing neuronal cell death and protecting against reperfusion injury [115,117,125]. Moreover, both resveratrol and curcumin appear to promote the shift in polarisation of microglia from the pro-inflammatory M1 phenotype to the anti-inflammatory M2 functional phenotype, which is responsible for tissue repair [62,116]. Both phytochemicals improve mitochondrial membrane potential and decrease cytochrome c release [55,59,89,129].

### 8.2. Neuroprotective Mechanisms Reported Only for Resveratrol

Despite similarities, several mechanisms of action seem unique for each of the two compounds. One of resveratrol’s distinctive properties is the activation of the sirtuin signaling network, particularly SIRT1 and SIRT3. This effect is responsible for increased ischaemic tolerance of the neuronal tissue and may be related to increased levels of BDNF and downregulation of UCP2 [58,74,75,76]. Another unique aspect of resveratrol is its extensive interaction with the Sonic Hedgehog signalling pathway, contributing to synaptic formation, neurite outgrowth, and neural stem cell proliferation, as well as the regulation of microglial inflammatory response [57,58,99,100]. Furthermore, resveratrol has been reported to stimulate angiogenesis through increased VEGF and MMP-2 expression and preserve BBB integrity by suppressing SUR1 and AQP-4 expression [63,64]. Clinical studies also suggest that resveratrol may enhance the efficacy of thrombolytic therapy with tissue plasminogen activator and improve long-term control of vascular risk factors following stroke [102,103].

### 8.3. Neuroprotective Mechanisms Reported Only for Curcumin

Curcumin has more evidence supporting its effects on microRNA modulation, with the available research identifying miR-7-5p and miR-1287-5p as important contributors to the compound’s neuroprotective properties, leading to reduced oxidative stress and inhibiting apoptosis [109,110,111]. Additionally, the anti-inflammatory effect of curcumin seems to be related to decreased signalling via the TLR4/p38/MAPK pathway [117]. Prolonged administration has been associated with enhanced neurogenesis through activation of Wnt/β-catenin signalling and increased expression of neurogenic transcription factors, including NeuroD1, Pax6, and Ngn2 [133]. This effect seems particularly interesting for its potential application in post-stroke regeneration and long-term neurological recovery. Moreover, curcumin appears to preserve neurotransmitter homeostasis by restoring dopamine, norepinephrine, and serotonin levels following cerebral ischaemia [128].

Overall, both compounds exhibit highly pleiotropic mechanisms that target many of the same pathological processes involved in stroke. However, resveratrol appears to exert greater effects on ischaemic preconditioning, angiogenesis, and vascular protection, suggesting it may have potential applications in prophylaxis of primary and recurrent stroke. Curcumin, on the other hand, demonstrates stronger modulation of autophagy, neurogenesis, and neurotransmitter regulation, which may play a greater role in long-term post-ischaemic neurological recovery.

## 9. Conclusions and Future Perspectives

Polyphenols, particularly resveratrol and curcumin, represent promising candidates for adjunctive therapy and prevention in stroke because of their broad anti-inflammatory, antioxidant, anti-apoptotic, and neuroprotective properties. The available evidence indicates that both compounds modulate multiple molecular pathways involved in ischaemic and haemorrhagic brain injury, including oxidative stress, neuroinflammation, mitochondrial dysfunction, apoptosis, autophagy, and BBB disruption. In addition, they appear to promote neurogenesis, angiogenesis, microglial polarization towards a reparative phenotype, and functional recovery after cerebral injury. Recent findings further suggest that some of these effects may be mediated through interactions with the gut microbiota. Resveratrol and curcumin can modify microbial composition, intestinal barrier integrity, and the production of microbiota-derived metabolites relevant to cerebrovascular health, including short-chain fatty acids, trimethylamine N-oxide precursors, and bile acid derivatives. By limiting intestinal dysbiosis, strengthening epithelial tight junctions, and reducing the translocation of bacterial products, these polyphenols may attenuate systemic inflammation and secondary neuroinflammatory responses after stroke. The gut microbiota also participates in the biotransformation of resveratrol and curcumin into biologically active metabolites, indicating that their effects are bidirectional and may depend partly on individual microbiome composition. However, the causal contribution of these microbiota-dependent pathways to stroke prevention and recovery has not yet been directly established. A substantial body of preclinical data from in vitro and animal studies shows that resveratrol and curcumin can reduce infarct volume, attenuate brain oedema, preserve neuronal viability, and improve neurological and cognitive outcomes. Their beneficial effects in animal models of stroke have been observed both when administered prophylactically and after stroke onset, suggesting potential value in both prevention and treatment. Nevertheless, the interpretation of post-stroke neuroprotection requires particular caution. Polyphenols cannot be expected to restore tissue that has already undergone irreversible necrosis within the infarct core. Ischemic stroke, however, is a dynamic process in which cerebral blood flow may remain partially preserved through collateral circulation, particularly within the ischemic penumbra, while spontaneous or therapeutic recanalization may subsequently restore perfusion to previously ischemic regions. Accordingly, circulating resveratrol, curcumin, or their metabolites may still reach viable peri-infarct and reperfused tissue. Importantly, restoration of cerebral blood flow does not immediately terminate tissue injury. Reperfusion itself may contribute to oxidative stress, inflammatory signaling, mitochondrial dysfunction, blood–brain barrier disruption, and secondary neuronal damage. The potential post-stroke effects of resveratrol and curcumin should therefore be interpreted primarily as an ability to limit secondary injury and the progression of tissue damage in salvageable or reperfused regions, rather than as an ability to rescue irreversibly necrotic tissue. Their pleiotropic actions may additionally influence the cerebral vasculature, endothelial function, BBB integrity, immune responses, and other systemic and local mechanisms involved in ischemia–reperfusion injury. Preclinical studies also suggest that these polyphenols may complement established or emerging therapeutic approaches, including thrombolysis, endovascular intervention, stem cell-based strategies, and selected pharmacological treatments. Such findings support the concept that multimodal compounds may be particularly attractive in a complex and heterogeneous disorder such as stroke. However, the predictive value of experimental models remains limited. Commonly used animal models reproduce selected aspects of human cerebral ischemia but do not fully reflect the age, comorbidities, vascular heterogeneity, variable collateral circulation, and incomplete or delayed reperfusion commonly observed in patients. Consequently, favorable experimental findings should be regarded as mechanistically informative and hypothesis-generating rather than as direct evidence of clinical efficacy.

Despite the encouraging preclinical findings, neither resveratrol nor curcumin can currently be regarded as a validated adjunctive treatment for stroke. Available clinical evidence remains limited and preliminary, while clinical translation is further constrained by poor solubility, low systemic bioavailability, rapid metabolism, uncertainty regarding optimal dosing and timing, and substantial interindividual variability. These pharmacokinetic limitations may not necessarily preclude biological activity, particularly given the potential contribution of active metabolites and gastrointestinal or microbiota-mediated mechanisms, but they complicate dose selection and interpretation of systemic exposure. Therefore, future research should follow a stepwise validation strategy aimed at determining whether these phytochemicals can realistically be incorporated into stroke management. First, additional preclinical studies should directly compare free compounds, structurally modified analogues, nanoformulations, and exosome-based delivery systems using standardized stroke models, clinically relevant doses, and clearly defined therapeutic windows. These studies should evaluate not only infarct volume and neurological outcomes, but also pharmacokinetics, BBB penetration, brain tissue concentrations, haemorrhagic risk, and interactions with established therapies such as thrombolysis, endovascular thrombectomy, antiplatelet agents, anticoagulants, antihypertensive drugs, and statins. Second, early-phase clinical trials should evaluate resveratrol and curcumin separately, rather than as a combined resveratrol-curcumin intervention. Phase I/II studies should focus on safety, tolerability, pharmacokinetic profiles, target engagement, and biomarker responses in clearly defined patient populations, including patients after ischaemic stroke, patients after haemorrhagic stroke, and individuals requiring secondary prevention. Particular attention should be given to hepatic and renal safety, bleeding risk, drug–drug interactions, and feasibility of administration during the acute and subacute phases of stroke. Third, randomized placebo-controlled trials should determine whether adjunctive resveratrol or curcumin improve clinically meaningful outcomes when added to standard care. These trials should be stratified according to stroke subtype, treatment timing, and baseline vascular risk. Primary and secondary outcomes should include participants’ functional status, cognitive recovery, quality of life, recurrent vascular events, mortality, infarct volume or haematoma evolution on neuroimaging, and circulating biomarkers of inflammation, oxidative stress, endothelial injury, and BBB disruption. Finally, if safety and preliminary efficacy are confirmed, larger multicentre trials should compare optimized formulations of each compound against placebo as adjuncts to guideline-based stroke therapy. Such studies should establish whether resveratrol or curcumin provide added value in acute neuroprotection, post-stroke recovery, or long-term secondary prevention. Overall, resveratrol and curcumin remain promising but investigational candidates for adjunctive stroke therapy. Their future clinical relevance will depend on rigorous pharmacological optimization and well-designed translational studies capable of linking experimental neuroprotection with measurable patient benefit.

## Figures and Tables

**Table 1:** Combined results from the in vivo studies concerning resveratrol’s role in prevention and treatment of stroke.

Dosage	Animal Species	Stroke Model	Way of Administration	Time Period	Results	Ref.
30 mg/kg	Male wild-type C57BL/6 mice	MCAO	Oral administration (1 dose per day for 3 days preceding the onset of ischemia period)	After ischemia period, a reperfusion period started.	↑ neuronal function ↓ motor deficits ↓ infarct volume	[61]
100 mg/kg	C57/BL male adult mice	MCAO	Intraperitoneal injections, made at various time points (0, 8 and 18 h) after the induction of ischemia period.	After 2 h of ischemia period, the arteries were unblocked, starting a reperfusion period.	↓ miR-155 expression ↑ M2 microglial polarization ↓ neuroinflammation ↑ activation of BV2 microglia	[62]
50 mg/kg	Adult male Balb/c mice	MCAO	Oral administration (1 dose per day until day 7, with first dose applied at various time points (5 min before or 24 h after or 72 h after the onset of ischemia period)	After 2 h of ischemia period, the arteries were unblocked, starting a 7-day-long reperfusion period after which the animals were sacrificed.	Beginning of administration 5 min before MCAO: ↓ neurologic scores ↓ infarct volume ↑ microvessels formation ↑ MMP-2 and VEGF mRNA levels β-actin mRNA unchanged Beginning of administration 72h after MCAO: ↓ VEGF protein β-actin protein levels unchanged	[63]
1.9 mg/kg	Male Wistar rats	MCAO	Single intravenous injection made at the reperfusion onset	After 2 h of ischemia period, the arteries were unblocked, starting a 24 h long reperfusion period after which the animals were sacrificed.	↓ neurological deficits ↓ infarct volume ↑ protection of brain tissue ↓ Sp1 activation ↓ overexpression of SUR1 and Abcc8 ↓ overexpression of AQP4	[64]
25 mg/kg	Male Wistar rats	MCAO	The single-mild-stroke group: 1 dose per day, administered orally, for 3 days preceding the onset of ischemia period The recurrent-stroke groups: 1 dose per day, administered orally for 3 days, with first dose applied 1 h after the first stroke and last dose 1 h before the second stroke	In single-mild-stroke group 30 min after the onset of ischemia period, the arteries were unblocked, starting a reperfusion period. In the recurrent-stroke group after 3 days of first reperfusion period, rats received second transient MCAO.	↓ neuronal cells death ↓ neuroinflammation ↓ oxidative stress ↓ BBB disruption ↓ cerebral oedema without affecting cerebral blood flow	[65]
30 mg/kg	C57BL/6J male mice	ICH	Intraperitoneal injections performed every day for 3 days preceding the onset of ICH and 7 days after	7 days after the onset of ICH the animals were sacrificed.	↑ neurological function ↓ neuronal cells death ↑ hematoma clearance ↓ activation of CD16+ M/Ms ↓ TNF levels	[66]
20 mg/kg	Male Wistar rats	MCAO	Intraperitoneal injections, made every day, for 21 days preceding the onset of ischemia period	After 2 h of ischemia period, the arteries were unblocked, starting a 24 h long reperfusion period after which the animals were sacrificed.	↑ motor function ↓ infarct volume ↓ MDA levels ↓ GSH levels	[67]
30 mg/kg	Adult male Sprague–Dawley rats	MCAO	Intraperitoneal injections performed every day for 7 days and once again 30 min preceding the onset of ischemia period	After 2 h of ischemia period, the arteries were unblocked, starting a reperfusion period. Depending on the subgroup, the rats were sacrificed at various time points (2, 6 and 24 h after onset of reperfusion).	↑ neurological scores ↓ infarct volume ↓ brain water content ↓ MDA levels ↑ SOD activity ↑ NRF2 and HO-1 expression ↓ caspase-3 expression	[68]
20 mg/kg	Wild-type mice	MCAO	Oral administration (1 dose 1 h before the onset of ischemia period or 1 dose per day for 3 days preceding the onset of ischemia period)	Mice were sacrificed 24 h after the induction of ischemia period.	↓ infarct volume	[69]
50 mg/kg	Adult male Sprague–Dawley rats or wild-type C57Bl/6J mice	MCAO	Single intraperitoneal injection (2 days before the onset of ischemia period)	Animals were sacrificed 24 h or 7 days after the induction of ischemia period.	↑ neuroprotection ↓ infarct volume ↑ neurological scores ↓ cholinergic cell loss in the medial septal nucleus and diagonal band of Broca ↑ cognitive impairments ↑ hippocampal long-term potentiation	[70]
30 mg/kg	Adult male Sprague–Dawley rats	MCAO	Intraperitoneal injections, performed every day for 7 days and once again preceding the onset of ischemia period	After 2 h of ischemia period, the arteries were unblocked, starting a 24 h long reperfusion period after which the animals were sacrificed.	↑ neurological function ↓ infarct volume ↓ neuronal damage ↓ neuronal apoptosis ↑ the expression of p-JAK2, p-STAT3, p-AKT, p-mTOR, and BCL-2 ↓ cleaved caspase-3 and Bax levels	[71]
30 mg/kg	Adult male Sprague–Dawley rats	MCAO	Intraperitoneal injections performed every day for 7 days and once again preceding the onset of ischemia period	After 1 h of ischemia period, the arteries were unblocked, starting a 24 h long reperfusion period after which the animals were sacrificed.	↑ neuronal viability ↓ infarct volume ↑ neurological function ↑ Shh, Ptc-1, Smo, and Gli1 expression	[72]
30 mg/kg	Adult male C75 black mice	MCAO	Intraperitoneal injections, made every day, for 7 days preceding the onset of ischemia period	After 1 h of ischemia period, the arteries were unblocked, starting a reperfusion period. Depending on the subgroup, the rats were sacrificed at various time points (24 h or 3 days after onset of reperfusion).	↓ infarct volume ↑ neurological function ↓ microglial activation ↓ TNF-α and IL-1β levels ↑ IL-10 level ↑ Shh, Ptc-1, Smo, and Gli-1 expression and ↑ Smo/Gli-1 translocation	[73]
100 mg/kg	Sprague–Dawley rats	ACA	Single intraperitoneal injection (48 h before ACA)	After 8 min of ACA period, resuscitation was initiated, starting a 7-day-long reperfusion period, after which the animals were sacrificed.	↑ Sirt1 activity in the hippocampus ↓ UCP2 levels ↑ the ADP/O ratio in hippocampal mitochondria ↑ ATP synthesis efficiency	[74]
10 mg/kg	Male C57Bl/6J mice	MCAO	Intraperitoneal injections, made at various frequencies (every other day, every day, intermittent, single) for 14 days before the ischemia period onset	Mice were sacrificed 24 h after the induction of ischemia period.	↓ infarct volume ↑ Sirt1 protein levels ↓ synaptic uncoupling protein 2 levels ↑ cortical BDNF levels	[75]
20 mg/kg	Male Sprague–Dawley rats	MCAO	Single intraperitoneal injection (30 min before the onset of ischemia period)	After 1 h of ischemia period, the arteries were unblocked, starting a reperfusion period.	↑ miR-149-5p activity ↑ Sirt1 activation ↑ p53 and caspase-3 activity	[76]
50 mg/kg	Male rats	MCAO	Single intraperitoneal injection (2 days before the onset of ischemia period)	After 1.5 h of ischemia period, the arteries were unblocked, starting a 24 h long reperfusion period, after which the animals were sacrificed.	↓ neuronal loss ↓ in total PARP1 protein ↑ levels of NAD+/NADH ↓ AIF nuclear translocation	[78]
Up to 0.05% of food ration mass	SHRSP	-	Oral administration (resveratrol diet for 1 week)	After 1 week of resveratrol diet rats were sacrificed or the onset of stroke was evaluated.	There was no significant delay in time to onset of the stroke or prolonged lifespan in SHRSP	[82]
2 × 10^−3^ mg/kg 2 × 10^−5^ mg/kg with LA (0.005 mg/kg)	Male Sprague–Dawley rats	MCAO	Intravenous injections, made at various time points (30 min before or 15 min after the onset of ischemia period, and 0, 30, 60, 90 min after reperfusion).	First group: 30 min of ischemia period followed by a 5.5 h long reperfusion period, after which the animals were sacrificed. Second group: 6 h of ischemia period, after which the animals were sacrificed.	Resveratrol pretreatment: ↓ infarct volume (dose-dependent), with significant reduction at the highest doses. Neuroprotection was observed when resveratrol was administered up to 60 min post-reperfusion Combined pretreatment with resveratrol and LA: ↓ infarct volume dose-dependently Combined resveratrol and LA 30 min before tMCAO: ↓ apoptotic markers	[83]

Abbreviations: ACA, asphyxial cardiac arrest; ADP/O, adenosine diphosphate/oxygen; AIF, apoptosis inducing factor; AQP4, aquaporin-4; ATP, adenosine triphosphate; BBB, blood–brain barrier; BCL-2, B-cell lymphoma 2; BDNF, brain-derived neurotrophic factor; CD, cluster of differentiation; Gli-1, glioma associated oncogene-1; GSH, glutathione synthetase;HO-1, heme oxygenase-1; ICH, intracerebral hemorrhage; IL-10, interleukin-10; IL-1β, interleukin-1 β; JAK2, Janus kinase-2; LA, lactic acid; M/Ms, microglia/macrophages; MCAO, middle cerebral artery occlusion; MDA, malonyldialdehyde; miR-149-5p, microRNA-149-5p; MMP-2, metaloproteinase-2; NAD+, nicotinamide adenine dinucleotide cation; NADH, nicotinamide adenine dinucleotide; NRF2, nuclear factor erythroid 2-related factor 2;p53, protein-53; p-AKT, phosphorylated AKT; PARP1, poly(ADP-ribose) polymerase 1; p-mTOR, phosphorylated mammalian target of rapamycin; p-STAT3, phosphorylated signal transducer and activator of transcription-3; Ptc-1, patched protein-1; Shh, sonic hedgehog; SHRSP, stroke-prone spontaneously hypertensive rats; Sirt1, sirtuin-1; Smo, smoothened receptor; SOD, superoxide dismutase; SUR1, sulfonylurea receptor-1; tMCAO, transient middle cerebral artery occlusion; TNF-α, tumor necrosis factor α; UCP2, uncoupling protein 2; VEGF, vascular endothelial growth factor. Notation: ↑ means upregulation/increase; ↓ means downregulation/decrease.

**Table 2:** Combined results from studies concerning molecular alterations of resveratrol particles to improve their bioavailability and effectiveness in target tissues.

Type of Modification	Uptake Strategy	BBB Penetration	Key Biological Effects	Comparison to Unmodified Resveratrol	Ref.
Nanoencapsulation of resveratrol in self-assembled PVP-b-PCL polymeric nanoparticles	Enhanced neuronal endocytosis	Not mentioned	↓ LDH release ↓ ROS activity ↓ MDA levels ↓ apoptosis-related markers	↑ neuronal uptake ↑ protection against oxidative stress ↑ controlled release	[84]
Encapsulation in polysorbate-stabilized nanostructured lipid carriers	Passive membrane permeation enabled by nanoscale lipid carriers with high drug encapsulation efficiency	Enhanced penetration and brain targeting	↑ neuronal uptake ↑ anti-apoptotic activity ↑ antioxidant activity ↓ neuronal apoptosis ↓ neuroinflammation	↑ brain delivery 80-times greater neuroprotective potency	[85]
Nanoencapsulation of resveratrol in mesoporous silica nanoparticles, PLA gatekeeper coating, LDLR-peptide surface conjugation	LDLR-peptide-mediated receptor transcytosis across brain endothelial cells; PLA shell acts as a ROS-responsive gatekeeper, degraded by microglial superoxide to trigger release	Enhanced penetration via LDLR-mediated transcytosis in vitro (not tested in vivo)	↑ delivery to inflamed CNS tissue ↓ microglial activation ↓ superoxide production ↓ NO production ↓ p47phox ↓ Iba-1 ↓ iNOS	↑ receptor-targeted brain delivery ↑ bioavailability ↑ BBB permeability ↑controlled release	[86]
Nanoencapsulation of resveratrol in pH-responsive PEG-acetal-PCL-PEG micelles, dual-functionalized with cRGD and TPP	cRGD-mediated BBB transcytosis, followed by lysosomal acid-triggered PEG shedding and TPP-mediated mitochondrial localization	Enhanced BBB penetration via cRGD-mediated transcytosis through overexpressed αvβ3 integrin	↑ mitochondrial protection ↑ activation of the SIRT1/PGC-1α/NRF1/TFAM pathway ↑ polarization of microglia from the pro-inflammatory M1 to the anti-inflammatory M2 phenotype ↑ anti-inflammatory cytokines ↑ long-term cognitive recovery ↑ overall survival ↓ pro-inflammatory cytokines ↓ BBB leakage ↓ ROS production	↑ motor and cognitive recovery ↑ delivery efficiency ↑ intracellular targeting ~4.7-fold greater brain accumulation ↑ M2/M1 microglial polarization ↓ infarct size ↓ apoptosis ↓ neutrophil infiltration	[87]
Nanoencapsulation in amphiphilic SAOR micelles, camouflaged with macrophage-membrane vesicles	-Macrophage membrane camouflage exploits macrophage homing to the I/R lesion -ROS(H2O2) cleavable oxalate linker triggers site-specific release	Macrophage membrane camouflage enables BBB-crossing ability	↑ neurological recovery ↓ infarct volume ↓ apoptosis	↑ cell viability under oxidative stress ↑ preferential accumulation at injury site ↑ ROS-triggered controlled release	[88]
Malibatol A (naturally occurring resveratrol oligomer)	Systemic administration, not mentioned directly	Not directly mentioned; brain effects after intravenous administration imply penetration	↓ infarct volume ↓ neurological deficits ↑ mitochondrial membrane potential ↑ Bcl-2 ↓ Bax ↓ cytochrome c release ↓ caspase-9/-3 activity ↓ ROS ↓ 3-NT ↓ 4-HNE	Not compared	[89]
Covalent hybridization of resveratrol-derived stilbene fused to a tetramethylpyrazine core	Hybrid molecule’s intrinsic properties	Not mentioned.	↓ platelet aggregation ↑ antioxidant protection ↑ neuroprotection	↑ Anti-platelet aggregation potency ↑ antioxidant protection in PC12 cells	[91]

Abbreviations: 3-NT, 3-nitrotyrosine; 4-HNE, 4-hydroxynonenal; Bax, BCL-2-associated X protein; Bcl-2, B-cell lymphoma 2; CNS, central nervous system; cRGD, cyclo(L-arginine–glycine–L-aspartic acid–D-tyrosine–L-lysine); Iba-1, ionized calcium-binding adapter molecule 1; iNOS, inducible nitric oxide synthase; LDH, lactate dehydrogenase; LDLR, low-density lipoprotein receptor ligand peptide; MDA, malondialdehyde; MSAOR@Cur, macrophage membrane-camouflaged sialic acid–Angelica polysaccharide–oxalate–resveratrol/curcumin nanoparticles; NLCs, nanostructured lipid carriers; NO, nitric oxide; p47phox, phagocyte NADPH oxidase 47-kDa subunit; PC12 cells, rat pheochromocytoma cells; PEG-acetal-PCL-PEG, poly(ethylene glycol)-acetal-poly(ε-caprolactone)-poly(ethylene glycol); PLA, poly(lactic acid); PPAR, peroxisome proliferator-activated receptor; PVP-b-PCL, poly(N-vinylpyrrolidone)-block-poly(ε-caprolactone); RES-NPs, resveratrol-loaded PVP-b-PCL nanoparticles; ROS, reactive oxygen species; SAOR, sialic acid–Angelica polysaccharide–oxalate–resveratrol; SIRT1/PGC-1α/NRF1/TFAM pathway, sirtuin 1/peroxisome proliferator-activated receptor gamma coactivator 1-alpha/nuclear respiratory factor 1/mitochondrial transcription factor A pathway; TPP, triphenylphosphine. Notation: ↑ means upregulation/increase; ↓ means downregulation/decrease.

**Table 3:** Summarized reports from clinical trials assessing resveratrol’s efficacy in treatment of stroke and resulting disability.

Dosage	Type of Brain Injury	Participants	Way of Administration	Results	Ref.
2.5 mg/kg	ischemic stroke	*n* = 312; 54–75 years old; clearly defined time of onset of stroke; with a deficit measurable on the NIHSS, and with no evidence of intracranial hemorrhage in baseline CT scan of the brain	Oral administration (simultaneously with r-tPA, with 10% as a bolus followed by the remaining 90% as a constant infusion over 60 min.) according to onset-to-treatment time: an early-onset group (0–120 min) and a delayed-onset group (120–240 min).	In both the early- and delayed-onset groups, resveratrol significantly improved outcomes compared with tissue plasminogen activator alone. Resveratrol decreased MMP-2 and MMP-9 levels and lowered NIHSS scores.	[102]
100 or 200 mg/day	ischaemic or haemorrhagic stroke	*n* = 228; 55~75 years; first stroke in the last 12 months, clinically stabilized after stroke	Oral administration (12-month)	↓ BMI ↓ serum LDL cholesterol levels ↓ serum triglyceride levels ↓ serum glucose levels ↑ serum HDL cholesterol levels ↓ HbA1c (%)	[103]
500 ± 10 mg/day	ischemic stroke in the territory of the middle cerebral artery	*n* = 100; 45–90 years old; in acute stage, symptoms lasted more than 24 h	Oral administration (capsules contained 170 mg resveratrol, 3 capsules per day for 30 consecutive days)	Resveratrol and placebo group did not differ significantly in: SBP and DBP, NIHSS, the Barthel index, MRS	[104]

Abbreviations: BMI, body mass index; CT, computed tomography; DBP, diastolic blood pressure; HbA1c, glycated hemoglobin A1c; HDL, high-density lipoprotein; LDL, low-density lipoprotein; MMP-2, matrix metalloproteinase-2; MMP-9, matrix metalloproteinase-9; MRS, modified Rankin Scale; NIHSS, National Institutes of Health Stroke Scale; r-tPA, recombinant tissue plasminogen activator; SBP, systolic blood pressure. Notation: ↑ means upregulation/increase; ↓ means downregulation/decrease.

**Table 4:** Combined results from the in vivo studies concerning curcumin’s role in prevention and treatment of stroke.

Dosage	Animal Species	Stroke Model	Way of Administration	Time Period	Results	Ref.
40 mg/kg	Adult male Sprague–Dawley rats with diabetes	Right-side MCAO	Oral administration (1 dose after the onset of ischemia period)	After 90 min of ischemia period, the arteries were unblocked. Rats were sacrificed at specific time points (6 h, 12 h, 24 h, 72 h) after reperfusion.	↑ neurological function, ↑ expression of GLUT1, GLUT3 and Bax, ↓ cerebral infarct volume, ↓ brain edema, ↓ apoptotic cells, ↓ Bax expression	[114]
25 mg/kg	Male albino rats	MCAO	Single intraperitoneal injection	After 30 min of ischemia period, the arteries were unblocked and the rats were sacrificed	↑ Bcl-2, SIRT1, expression, ↑ MMP activity, ↑ neurological scores, ↓ p53 and Bax expression, ↓ TNF-α, IL-6 and ROS levels, ↓ infarct volume, ↓ brain water content	[115]
150 mg/kg	Male C57BL/6 mice	Distal MCAO with BCCAO	Intraperitoneal injections, made at the start of the reperfusion period and again after 24 h.	Rats were sacrificed 72 h after the induction of ischemia period	↑ Arg-1, YM1/2 expression ↑ CD206 + Iba1+ M2 microglioma/macrophages ↑ sensorimotor function ↓ CD32 expression ↓ CD16 + Iba1+ M1 microglia/macrophages ↓ cerebral ischemic damage ↓ IFN-γ-induced M1 polarization ↓ TNF-α, IL-6, IL-12p70, iNOS levels	[116]
200 mg/kg	Male Sprague Dawley rats	Intraluminal MCAO	Single intraperitoneal injection (30 min after the onset of ischemia period)	After 2 h of ischemia period, arteries were unblocked, starting a 22 h long reperfusion period after which the animals were sacrificed	↑ p-mTOR, p-Akt expression ↑ neurological function ↓ TNF-α, IL-6, IL-1 levels ↓ p-38, TLR4, LC3-II/LC3-I expression ↓ iNOS levels ↓ autophagy via the PI3K/Akt/mTOR pathway ↓ inflammation via the TLR4/p38/MAPK pathway ↓ brain damage	[117]
100 mg/kg	Mice	MCAO	Single intraperitoneal injection (15 min after the onset of ischemia period)	Rats were sacrificed 24 h after the induction of ischemia period	↑ neuroprotection ↑ neurogenesis	[118]
50 mg/kg	Sprague–Dawley rats	MCAO	Single intraperitoneal injection (1 h after the onset of ischemia period)	Rats were sacrificed 24 h after the induction of ischemia period	↑ adenosylhomocysteinase, ICDH, UCH-L1, PLPP levels ↑ eIF4A expression ↑ neuroprotection	[119]
300 mg/kg (dissolved in 1.0 mL corn oil)	Wistar rats	MCAO	Single intraperitoneal injection (30 min after the onset of ischemia period)	After 1 h of ischemia period, arteries were unblocked, starting a 24 h long reperfusion period after which the animals were sacrificed	↑ NRF2 expression ↓ brain water content ↓ infarct volume ↓ NF-κB expression ↓ MDA levels	[121]
100 mg/kg	Male Sprague-Dawley rats	Right side MCAO	Single intraperitoneal injection (15 min after the onset of ischemia period)	Rats were sacrificed 24 h after the induction of ischemia period	↑ NRF2 and HO-1 expression ↓ neurological deficits ↓ infarct size ↓ brain water content	[122]
300 mg/kg	Male Sprague–Dawley rats	Focal embolic model	Single intraperitoneal injection (4 h after the onset of ischemia period)	Rats were sacrificed 24 h after the induction of ischemia period	↑ GSH activity ↑ sensory–motor function ↓ peroxynitrite, NO, ROS, iNOS, nitrate, nitrite levels ↓ GSH-Px loss ↓ neutrophil infiltration ↓ neurological deficits ↓ edema volume ↓ infarct size	[123]
300 mg/kg	Adult male Sprague-Dawley rats	intraluminal MCAO	Single intraperitoneal injection (1 h after the onset of ischemia period)	After 2 h of ischemia period, arteries were unblocked, starting a 24 h long reperfusion period after which the animals were sacrificed	↓ caspase-3 protein expression ↓ TUNEL-positive cells ↓ neuronal injury ↓ neurological deficits ↓ infarct size	[124]
500 mg/kg	Sprague-Dawley rats	MCAO/R	Single intraperitoneal injection (1 h after the onset of ischemia period)	After 1 h of ischemia period, arteries were unblocked, starting a 24 h long reperfusion period after which the animals were sacrificed	↑ mitochondrial Bcl-2 expression ↑ anti-apoptotic mechanisms activation ↓ infarct volume ↓ MDA levels ↓ caspase-3, cytochrome c expression ↓ oxidative stress	[125]
100 mg/kg	Male SHRSP rats	-	Oral administration (1 dose per day until the death of each animal)	Rats were sacrificed 4 weeks after the induction of ischemia period	↑ time to stroke onset ↑ survival time ↑ carotid artery relaxation to ACh and SNP ↑ plasma nitrate and nitrite levels ↑ SOD activity ↑ UCP2 mRNA expression in carotid arteries ↑ cell viability ↑ NO production ↓ ROS, MDA levels	[126]
200 mg/kg	Male Wistar albino rats	Spinal cord I/R injury induced by occlusion of the abdominal aorta	Intraperitoneal injections, made every day, for 7 days before the onset of ischemia period	Rats were sacrificed 24 h after the induction of ischemia period	↑ locomotor function ↑ antioxidant defense mechanisms ↓ inflammatory cytokine levels ↓ oxidative stress ↓ lipid peroxidation ↓ apoptosis	[127]
50 mg/kg	Male Wistar rats	Global cerebral ischemia by left common carotid artery occlusion	Oral administration (1 dose 1 h before and 24 h after onset of ischemia) Oral administration (1 dose 1 h before and 1 dose per day for 7 days after the onset of ischemia period)	After 30 min of ischemia period, the arteries were unblocked and rats were sacrificed 1 h after the final drug administration After 30 min of ischemia period, the arteries were unblocked. Rats were sacrificed 24 h after the final drug administration	↑ 5-HT expression ↑ NE levels ↑ neuronal viability ↓ immunoreactivity for COX-2 ↓ TNF-α levels	[128]
50 mg/kg	Male Sprague–Dawley rats	transient MCAO	Intraperitoneal injections, made every day, for 5 days before the onset of ischemia period	After 2 h of ischemia period, arteries were unblocked, starting a 24 h long reperfusion period after which the animals were sacrificed	↑ MMP activity ↑ SIRT1 and Bax expression ↑ mitochondrial complex I activity ↑ mitochondrial cytochrome c levels ↑ neurological function ↓ Ac-p53 and Bac expression ↓ cytosolic cytochrome c levels ↓ TNF-α and IL-6 concentrations ↓ brain edema ↓ infarct volume	[129]
100 mg/kg	Male Wistar albino rats	MCAO	Oral administration (1 dose per day for 5 days preceding and 3 days after the onset of ischemia period)	After 2 h of ischemia period, arteries were unblocked, starting a 72 h long reperfusion period after which the animals were sacrificed	↑ SOD activity ↑ neurobehavioral performance ↓ mortality ↓ infarct area ↓ intracellular calcium levels ↓ lipid peroxidation	[130]
300 mg/kg	Older rats	BCCAO	Oral administration (1 dose per day for 21 days) and single intraperitoneal injection performed right after the induction of the ischemia period	After 30 min of ischemia period, arteries were unblocked, starting a 72 h long reperfusion period after which the animals were sacrificed	↑ GSH-Px, SOD, CAT activity ↑ Neurological scores ↓ MDA levels ↓ TNF-α, IL-6 concentration ↓ apoptotic index ↓ TUNEL-positive cells	[132]
100 mg/kg	Male C57BL/6J mice	BCCAO	Intraperitoneal injections, made every day, for 7 days after the onset of ischemia period	Mice were sacrificed 7 days after the induction of ischemia period	↑ Ngn2, Pax6, NeuroD1 expression ↑ Wnt/β-catenin signaling pathway proteins ↑ differentiation and maturation of newly generated neural cells ↑ NSC proliferation ↓ cognitive impairments	[133]
50 mg/kg	Male Wistar rats	4VOI	Oral administration (1 dose per day for 7 days after the onset of ischemia period)	Mice were sacrificed 7 days after the induction of ischemia period	↑ memory preservation, ↑ ACh levels, ↓ hippocampal neuronal apoptosis, ↓ memory deficits	[134]

Abbreviations: 4VOI, four vessel occlusion brain ischemia; 5-HT, 5-hydroxytryptamine; ACh, acetylcholine; Arg-1, arginase-1; Bax, Bcl-2-associated protein; BCCAO, bilateral common carotid artery occlusion; Bcl-2, B-cell lymphoma; CAT, catalase; CD, cluster of differentiation; COX-2, cyclooxygenase 2; eIF4A, eukaryotic initiation factor-4A; GLUT1, glucose transporter 1; GLUT3, glucose transporter 3; GSH, glutathione synthetase; HO-1, heme oxygenase 1; ICDH, isocitrate dehydrogenase; IFN-γ, interferon γ; IL-6, interleukin 6; iNOS, inducible nitric oxide synthase; I/R, ischemia/reperfusion; MAPK, mitogen-activated protein kinase; MCAO, middle cerebral artery occlusion; MCAO/R, middle cerebral artery occlusion/reperfusion; MDA, malonyldialdehyde; MMP, metalloproteinase; NE, norepinephrine; NeuroD1, neurogenic differentiation factor 1; NF-κB, nuclear factor kappa-light-chain-enhancer of activated B cells; Ngn2, neurogenin 2; NO, nitric oxide; NRF2, nuclear factor erythroid 2-related factor 2; NSC, neural stem cells; p-38, protein 38; Pax6, paired box protein 6; PI3K, phosphoinositide 3 kinase; PLPP, phospholipid phosphatase; p-mTOR, phosphorylated mammalian target of rapamycin; ROS, reactive oxygen species; SHRSP, stroke-prone spontaneously hypertensive rats; SIRT1, sirtuin 1; SNP, sodium nitroprusside; SOD, superoxide dismutase; TLR4, toll-like receptor 4; TNF-α, tumor necrosis factor α; TUNEL, terminal deoxynucleotidyl transferase dUTP nick end labeling; UCH-L1, ubiquitin carboxy-terminal hydrolase L1; UCP2, uncoupling protein 2. Notation: ↑ means upregulation/increase; ↓ means downregulation/decrease.

**Table 5:** Combined results from studies concerning molecular alterations of curcumin particles to improve their bioavailability and effectiveness in target tissues.

Type of Modification	Uptake Strategy	BBB Penetration	Key Biological Effects	Comparison to Unmodified Resveratrol	Ref.
Nanoencapsulation in amphiphilic SAOR micelles, camouflaged with macrophage membrane vesicles	Macrophage membrane camouflage exploits macrophage homing to the I/R lesion. ROS(H2O2) cleavable oxalate linker triggers site-specific release.	Macrophage membrane camouflage enables BBB-crossing ability	↑ neurological recovery ↓ infarct volume ↓ apoptosis	↑ Cell viability under oxidative stress ↑ preferential accumulation at injury site ↑ ROS-triggered, controlled release	[88]
c(RGDyK)-conjugated functional ligands on exosomal surfaces using bio-orthogonal copper-free azide-alkyne cyclo-addition	c(RGDyK) conjugation significantly enhances the tropism to the integrin αvβ, a molecule expressed on reactive endothelial cells after ischemia	Improved ability to cross BBB	↑ Neuroprotective efficacy ↓ Neuroinflammation ↓ oxidative stress ↑ Ischemia lesion targeting	↑ suppression of the inflammatory response, ↓ cellular apoptosis, ↑ targeting delivery, ↓ TNF-α, ↓ IL-1β, ↓ IL-6, ↓ activation of microglia, ↓ p-p65 expression	[135]
Curcumin-loaded nanogel/nanoparticles prepared by gelation with tripolyphosphate	Passive BBB penetration attributed to the small particle size (~51 nm)	BBB crossing and brain biodistribution were not directly quantified or visualized	↑ motor functions ↑ muscle strength ↑ GPx activity ↑ GSH activity ↑ GST activity ↑ SOD activity ↑ CAT activity ↓ oxidative stress ↓ inflammation	Not directly compared	[136]
Nano-emulsion encapsulation in a castor-oil core stabilized by lecithin/PEG-660 stearate (lipid nanocarrier)	Passive exploitation of ischaemia-induced BBB disruption	lipid carrier crosses the disrupted BBB better than free curcumin	↓ haematoma area ↓ lipid peroxidation ↓ oxidative damage to protein ↑ neuroprotective efficacy ↑ post-stroke behavioural recovery ↓ post-stroke weight loss	↑ bioavailability in brain ↑ sustained release ↑ behavioral recovery ↓ oxidative damage ↓ haematoma area	[137]
Covalent conjugation to an ONOO^−^-cleavable self-immolative chemiluminescent linker (Cur-CL), co-encapsulated with AIE-BODIPY (BDP-4) in a lactoferrin-functionalized nanoparticle	Lactoferrin-receptor-mediated uptake into brain cell. Endogenous ONOO^−^ triggers disease-site-specific curcumin release.	improved lactoferrin-mediated neuronal uptake	↓ apoptosis ↑ survival rates ↓ neuroinflammation ↓ infarct volume ↑ distinguishing between injury and normal areas for endogenous ONOO− detection in the early stage of ischemia ↑ cognition of post-stroke mice	Not directly compared	[138]
Small-molecule hybridization of curcumin with cyclohexyl bisphenol A replacing curcumin’s labile β-diketone with pyrazole ring	Passive BBB diffusion via lipophilicity	Improved penetration	↑ post-stroke behavioral function ↓ COX-2 level ↓ 5-LOX expression ↑ BDNF ↑ CaMKIIα ↑ ORP150	↑ ATP maintenance ↑ stability ↑ neurotrophic activity ↑ protection against excitotoxicity	[139,140]

Abbreviations: 5-LOX, 5-lipoxygenase; AIE-BODIPY, aggregation-induced emission-active boron-dipyrromethene fluorophore; BBB, blood–brain barrier; BDNF, brain-derived neurotrophic factor; BDP-4, fourth-generation boron-dipyrromethene derivative; CaMKIIα, calcium/calmodulin-dependent protein kinase II alpha; CAT, catalase; COX-2, cyclooxygenase-2; c(RGDyK), cyclo(L-arginine–glycine–L-aspartic acid–D-tyrosine–L-lysine); Cur-Cl, curcumin–chemiluminescent linker conjugate; GPx, glutathione peroxidase; GSH, reduced glutathione; GST, glutathione S-transferase; IL-1β, interleukin-1 beta; IL-6, interleukin-6; ORP150, 150-kDa oxygen-regulated protein; PEG-660, poly(ethylene glycol) with an average molecular weight of 660 Da; p-p65, phosphorylated nuclear factor kappa B p65 subunit; ROS, reactive oxygen species; SAOR, sialic acid–Angelica polysaccharide–oxalate–resveratrol; SOD, superoxide dismutase; TNF-α, tumor necrosis factor α. Notation: ↑ means upregulation/increase; ↓ means downregulation/decrease.

## Data Availability

The data used to support the findings of this study are included in this article.

## Abbreviations

The following abbreviations are used in this manuscript:

3-NT	3-nitrotyrosine
4-HNE	4-hydroxynonenal
4VOI	four-vessel occlusion-induced brain ischaemia
5-HT	5-hydroxytryptamine
5-LOX	5-lipoxygenase
ACA	asphyxial cardiac arrest
ACh	acetylcholine
ADP	adenosine diphosphate
ADP/O	adenosine diphosphate-to-oxygen ratio
AIE-BODIPY	aggregation-induced emission-active boron-dipyrromethene fluorophore
AIF	apoptosis-inducing factor
AKT	protein kinase B
AMPK	5′-adenosine monophosphate-activated protein kinase
AQP4	aquaporin 4
Arg-1	arginase 1
ATP	adenosine triphosphate
Bax	BCL-2-associated X protein
BBB	blood–brain barrier
BCCAO	bilateral common carotid artery occlusion
Bcl-2	B-cell lymphoma 2
BDNF	brain-derived neurotrophic factor
BDP-4	fourth-generation boron-dipyrromethene derivative
BMI	body mass index
BP	blood pressure
BV2	murine microglial BV2 cell line
c(RGDyK)	cyclo(L-arginine–glycine–L-aspartic acid–D-tyrosine–L-lysine)
CAT	catalase
CD	cluster of differentiation
CD16	cluster of differentiation 16
CD32	cluster of differentiation 32
CD206	cluster of differentiation 206
CNB-001	curcumin-based neuroprotective compound 001
CNPs	curcumin nanoparticles
CNS	central nervous system
COX	cytochrome c oxidase
COX-2	cyclooxygenase 2
CRP	*C*-reactive protein
CT	computed tomography
Cur-Cl	curcumin–chemiluminescent linker conjugate
CYP	cytochrome P450
CYP1A2	cytochrome P450 family 1 subfamily A member 2
CYP2B6	cytochrome P450 family 2 subfamily B member 6
CYP2C9	cytochrome P450 family 2 subfamily C member 9
CYP2C19	cytochrome P450 family 2 subfamily C member 19
CYP2D6	cytochrome P450 family 2 subfamily D member 6
CYP3A4	cytochrome P450 family 3 subfamily A member 4
DBP	diastolic blood pressure
DNA	deoxyribonucleic acid
DR	dietary restriction
eIF4A	eukaryotic initiation factor 4A
ERK	extracellular signal-regulated kinase
ERK1/2	extracellular signal-regulated kinases 1 and 2
Gli-1	glioma-associated oncogene 1
GLUT1	glucose transporter 1
GLUT3	glucose transporter 3
GPx	glutathione peroxidase
GSH	reduced glutathione
GST	glutathione S-transferase
H_2_O_2_	hydrogen peroxide
HbA1c	glycated haemoglobin A1c
HDAC	histone deacetylase
HDACs	histone deacetylases
HDL	high-density lipoprotein
HO-1	haem oxygenase 1
HT22	mouse hippocampal neuronal HT22 cell line
hUC-MSCs	human umbilical cord-derived mesenchymal stem cells
I/R	ischaemia/reperfusion
Iba-1	ionized calcium-binding adapter molecule 1
ICDH	isocitrate dehydrogenase
ICH	intracerebral haemorrhage
IDH	isocitrate dehydrogenase
IFN-γ	interferon gamma
IL-1β	interleukin 1 beta
IL-6	interleukin 6
IL-10	interleukin 10
IL-12p70	interleukin 12 p70
IL-18	interleukin 18
iNOS	inducible nitric oxide synthase
INR	international normalized ratio
IPC	ischaemic preconditioning
JAK2	Janus kinase 2
LA	lipoic acid
LC3-II/LC3-I	microtubule-associated protein 1 light chain 3 II to light chain 3 I ratio
LDH	lactate dehydrogenase
LDL	low-density lipoprotein
LDLR	low-density lipoprotein receptor
LONP2	lon peptidase 2 peroxisomal
LPMSNPs	LDLR peptide-conjugated PLA-coated mesoporous silica nanoparticles
M/Ms	microglia/macrophages
M1	pro-inflammatory microglial phenotype
M2	anti-inflammatory microglial phenotype
MA	malibatol A
MAPK	mitogen-activated protein kinase
MCAO	middle cerebral artery occlusion
MCAO/R	middle cerebral artery occlusion/reperfusion
MDA	malondialdehyde
miR-149-5p	microRNA-149-5p
miR-155	microRNA-155
miR-1287-5p	microRNA-1287-5p
MMP	matrix metalloproteinase
MMP-2	matrix metalloproteinase 2
MMP-9	matrix metalloproteinase 9
mRNA	messenger RNA
mRS	modified Rankin Scale
MS-275	entinostat
MSAOR@Cur	macrophage membrane-camouflaged sialic acid–Angelica polysaccharide–oxalate–resveratrol/curcumin nanoparticles
MSNPs	mesoporous silica nanoparticles
mTOR	mammalian target of rapamycin
NAD^+^	oxidized nicotinamide adenine dinucleotide
NADH	reduced nicotinamide adenine dinucleotide
NADPH	reduced nicotinamide adenine dinucleotide phosphate
NE	norepinephrine
NeuroD1	neurogenic differentiation factor 1
NF-κB	nuclear factor kappa B
Ngn2	neurogenin 2
NIHSS	National Institutes of Health Stroke Scale
NLCs	nanostructured lipid carriers
NLR	neutrophil-to-lymphocyte ratio
NLRP3	NLR family pyrin domain-containing protein 3
NO	nitric oxide
NOS2	nitric oxide synthase 2
NRF1	nuclear respiratory factor 1
NRF2	nuclear factor erythroid 2-related factor 2
NSC	neural stem cell
OATP	organic anion-transporting polypeptide
OATP1B1	organic anion-transporting polypeptide 1B1
OATP1B3	organic anion-transporting polypeptide 1B3
OGD	oxygen–glucose deprivation
OGD/R	oxygen–glucose deprivation/reoxygenation
OM-MSCs	olfactory mucosa-derived mesenchymal stem cells
ONOO^−^	peroxynitrite
ORP150	150-kDa oxygen-regulated protein
p-AKT	phosphorylated protein kinase B
p-JAK2	phosphorylated Janus kinase 2
P-gp	P-glycoprotein
p-mTOR	phosphorylated mammalian target of rapamycin
p-p65	phosphorylated nuclear factor kappa B p65 subunit
p-STAT3	phosphorylated signal transducer and activator of transcription 3
p47phox	phagocyte NADPH oxidase 47-kDa subunit
p50	nuclear factor kappa B p50 subunit
p53	tumour protein p53
p65	nuclear factor kappa B p65 subunit
PARP-1	poly(ADP-ribose) polymerase 1
Pax6	paired box protein 6
PC12 cells	rat pheochromocytoma cells
PEG	poly(ethylene glycol)
PEG-660	poly(ethylene glycol) with an average molecular weight of 660 Da
PEG-acetal-PCL-PEG	poly(ethylene glycol)-acetal-poly(ε-caprolactone)-poly(ethylene glycol)
PGC-1α	peroxisome proliferator-activated receptor gamma coactivator 1 alpha
PI3K	phosphoinositide 3-kinase
PLA	poly(lactic acid)
PLPP	phospholipid phosphatase
PPAR	peroxisome proliferator-activated receptor
PPAR-α	peroxisome proliferator-activated receptor alpha
PPAR-β/δ	peroxisome proliferator-activated receptor beta/delta
PPAR-γ	peroxisome proliferator-activated receptor gamma
Ptc-1	patched 1
PVP-b-PCL	poly(N-vinylpyrrolidone)-block-poly(ε-caprolactone)
RelA	nuclear factor kappa B p65 subunit
RES-NPs	resveratrol-loaded PVP-b-PCL nanoparticles
RNA	ribonucleic acid
ROS	reactive oxygen species
RPC	repetitive resveratrol preconditioning
r-tPA	recombinant tissue plasminogen activator
SAH	S-adenosylhomocysteinase
SAOR	sialic acid–Angelica polysaccharide–oxalate–resveratrol
SBP	systolic blood pressure
SH-SY5Y	human neuroblastoma SH-SY5Y cell line
Shh	sonic hedgehog
SHRSP	stroke-prone spontaneously hypertensive rats
SIRT1	sirtuin 1
SIRT3	sirtuin 3
Smo	smoothened
SNP	sodium nitroprusside
SOD	superoxide dismutase
STAT3	signal transducer and activator of transcription 3
SUR1	sulfonylurea receptor 1
TFAM	mitochondrial transcription factor A
TLR4	toll-like receptor 4
tMCAO	transient middle cerebral artery occlusion
TNF-α	tumour necrosis factor alpha
TPP	triphenylphosphine
TUNEL	terminal deoxynucleotidyl transferase dUTP nick-end labelling
UCH-L1	ubiquitin carboxyl-terminal hydrolase L1
UCP2	uncoupling protein 2
VEGF	vascular endothelial growth factor
Ym1	chitinase-like protein 3
